# A novel requirement for DROSHA in maintenance of mammalian CG methylation

**DOI:** 10.1093/nar/gkx695

**Published:** 2017-08-03

**Authors:** Athanasia Stathopoulou, Jyoti B. Chhetri, John C. Ambrose, Pierre-Olivier Estève, Lexiang Ji, Hediye Erdjument-Bromage, Guoqiang Zhang, Thomas A. Neubert, Sriharsa Pradhan, Javier Herrero, Robert J. Schmitz, Steen K.T. Ooi

**Affiliations:** 1 Department of Cancer Biology, UCL Cancer Institute, London WC1E 6BT, UK; 2 Bill Lyons Informatics Centre, UCL Cancer Institute, London WC1E 6BT, UK; 3 New England Biolabs, 240 Country Road, Ipswich, MA 01938, USA; 4 Institute of Bioinformatics, University of Georgia, 120 East Green Street, Athens, GA 30602, USA; 5 Department of Biochemistry and Molecular Pharmacology, Skirball Institute, NYU School of Medicine, New York, NY 10016, USA; 6 Department of Genetics, University of Georgia, 120 East Green Street, Athens, GA 30602, USA

## Abstract

In mammals, faithful inheritance of genomic methylation patterns ensures proper gene regulation and cell behaviour, impacting normal development and fertility. Following establishment, genomic methylation patterns are transmitted through S-phase by the maintenance methyltransferase Dnmt1. Using a protein interaction screen, we identify Microprocessor component DROSHA as a novel DNMT1-interactor. *Drosha*-deficient embryonic stem (ES) cells display genomic hypomethylation that is not accounted for by changes in the levels of DNMT proteins. DNMT1-mediated methyltransferase activity is also reduced in these cells. We identify two transcripts that are specifically upregulated in *Drosha-* but not *Dicer*-deficient ES cells. Regions within these transcripts predicted to form stem–loop structures are processed by Microprocessor and can inhibit DNMT1-mediated methylation *in vitro*. Our results highlight DROSHA as a novel regulator of mammalian DNA methylation and we propose that DROSHA-mediated processing of RNA is necessary to ensure full DNMT1 activity. This adds to the DROSHA repertoire of non-miRNA dependent functions as well as implicating RNA in regulating DNMT1 activity and correct levels of genomic methylation.

## INTRODUCTION

In mammals, methylation at CG dinucleotides is required for the monoallelic expression of genes subject to genomic imprinting, transcriptional silencing of retrotransposons and X chromosome silencing in females (1). In animals, genomic methylation patterns are initially established by the *de novo* methyltransferases Dnmt3A and Dnmt3B (2) following two stages of methylation loss that occurs prior to implantation of the embryo (3) as well as during the migration of primordial germ cells (PGCs) (4). Following establishment, genomic methylation patterns are transmitted through S-phase by the maintenance methyltransferase DNA methyltransferase 1 (DNMT1). Given the importance of faithful inheritance of cytosine methylation, understanding how DNMT1 is correctly regulated is crucial. Normal DNMT1 function is achieved by a combination of its correct targeting (5–7), control of protein stability (8,9) and regulation of its methyltransferase activity (10). However, our understanding of the mechanisms involved and how they interact remains incomplete.

RNA has long been proposed to regulate DNA methylation (11). In the mammalian germ line, DNA methylation establishment depends on the biogenesis of a particular class of small RNAs termed piwi-interacting (piRNA) in prospermatogonial stem cells (12,13), but the mechanism linking these two processes remains unknown. Although piRNAs are believed to instruct where DNMTs are targeted, it is also possible that RNA may have a regulatory function controlling methyltransferase activity. DNMT1-interacting RNAs (DiRs) have recently been described (14) that are postulated to inhibit DNMT1 catalytic activity through their interaction with the C-terminal methyltransferase domain, although their general function and mechanisms regulating them are unknown.

Using a protein interaction screen to further define the mechanism by which DNMT1 is regulated, we identify the Microprocessor component DROSHA as a novel DNMT1-interactor. Using CRISPR/Cas gene editing to inactivate *Drosha* in mouse embryonic stem (ES) cells, we show that in its absence, genome-wide cytosine methylation is reduced and that DROSHArosha ensures full DNMT1 methyltransferase activity. We also present evidence demonstrating that human DROSHA is capable of processing regions of previously identified DiRs, and that these inhibit DNMT1-activity. Based on these results, we propose that DROSHA-mediated processing of DiRs is necessary to ensure full DNMT1 activity, adding to the DROSHA repertoire of non-miRNA dependent functions.

## MATERIALS AND METHODS

### Embryonic stem (ES) cell culture

Mouse ES cells were cultured in ES cell media that consisted of Dulbecco’s Modified Eagle’s Medium (DMEM) supplemented with 15% fetal bovine serum (FBS), 100 IU/ml penicillin, 100 mg/ml streptomycin, 2 mmol/l L-glutamine, MEM non-essential amino acids, 0.12 mmol/l β-mercaptoethanol and leukaemia inhibitory factor (LIF). During the targeting process, ES cells were cultured on mitomycin C-treated mouse embryonic fibroblasts (MEF) feeder cells. For downstream analysis, ES cells were cultured on gelatin-coated plates.

### Protein identification by nano-liquid chromatography coupled to tandem mass spectrometry (LC-MS/MS) analysis

Immunoaffinity-purified material from *Dnmt1^Tag/+^* and parental *Dnmt1^+/+^* ES cells were resolved briefly, using sodium dodecyl sulphate-polyacrylamide gel electrophoresis (SDS-PAGE), followed by staining with Coomassie Blue and excision of the separated protein bands. *In situ* trypsin digestion of polypeptides in each gel slice was performed as described (15). The tryptic peptides were purified using a 2 μl bed volume of Poros 50 R2 (Applied Biosystems, CA, USA) reversed-phase beads packed in Eppendorf gel-loading tips. The purified peptides were diluted to 0.1% formic acid and then subjected to nano-liquid chromatography coupled to tandem mass spectrometry (nano-LC-MS/MS) analysis as follows. Peptide mixtures (in 20 μl) were loaded onto a trapping guard column (0.3 × 5 mm Acclaim PepMap 100 C18 cartridge from LC Packings, Sunnyvale, CA, USA) using an Eksigent nano MDLC system (Eksigent Technologies, Inc. Dublin, CA, USA) at a flow rate of 20 μl/min. After washing, the flow was reversed through the guard column and the peptides eluted with a 5–45% acetonitrile gradient over 85 min at a flow rate of 200 nl/min, onto and over a 75-μ × 15-cm fused silica capillary PepMap 100 C18 column (LC Packings, Sunnyvale, CA, USA). The eluent was directed to a 75-μ (with 10-μ orifice) fused silica nano-electrospray needle (New Objective, Woburn, MA, USA). The electrospray ionization needle was set at 1800 V. A linear ion quadrupole trap-Orbitrap hybrid analyzer (LTQ-Orbitrap, ThermoFisher, San Jose, CA, USA) was operated in automatic, data-dependent MS/MS acquisition mode with one MS full scan (450–2000 m/z) in the Orbitrap analyzer at 60 000 mass resolution and up to 10 concurrent MS/MS scans in the Linear Trap Quadropole (LTQ) for the 10 most intense peaks selected from each survey scan. Survey scans were acquired in profile mode and MS/MS scans were acquired in centroid mode. The collision energy was automatically adjusted in accordance with the experimental mass (m/z) value of the precursor ions selected for MS/MS. Minimum ion intensity of 2000 counts was required to trigger an MS/MS spectrum; dynamic exclusion duration was set at 60 s.

Initial protein/peptide identifications from the LC-MS/MS data were performed using the Mascot search engine (Matrix Science, version 2.5.0; www.matrixscience.com) with the rodent segment of Uniprot protein database (20 255 sequences; European Bioinformatics Institute, Swiss Institute of Bioinformatics and Protein Information Resource). The search parameters were as follows: (i) two missed cleavage tryptic sites were allowed; (ii) precursor ion mass tolerance = 10 ppm; (iii) fragment ion mass tolerance = 0.8 Da; (iv) variable protein modifications were allowed for methionine oxidation, deriving cysteine acrylamide and protein N-terminal acetylation. MudPit scoring was typically applied using significance threshold score *P* < 0.01. Decoy database search was always activated and, in general, for merged LS-MS/MS analysis of a gel lane with *P* < 0.01, false discovery rate averaged ∼1%. Scaffold (Proteome Software Inc., Portland, OR), version 4_4_5 was used to further validate and cross-tabulate the MS/MS-based peptide and protein identifications. Protein and peptide probability was set at 95% with a minimum peptide requirement of 1.

### Western blot

Pelleted cells were lysed in Radioimmunoprecipitation (RIPA) buffer. Samples were quantified using Bio-Rad Protein Assay (Bio-Rad) and equal amounts of lysate were loaded onto Mini-Protean Gels (Bio-Rad). Following separation by electrophoresis, proteins were transferred onto nitrocellulose membrane (GE Healthcare), blocked in 1× Phosphate Buffered Saline + 0.1% Tween-20 (PBST) + 5% milk for 1 h before incubating with primary antibodies overnight. The next day, membranes were washed in PBST, incubated with secondary antibody, washed in PBST, before horseradish peroxidase (HRP) signal development using Luminata Forte Western HRP Substrate (Millipore). Signals were detected by exposure of membranes onto Hyperfilm ECL (Amersham).

### Antibodies

For western blot, the following antibodies were used at the dilutions indicated:

Anti-FLAG (1:3000, F3165, Sigma-Aldrich); Anti-myc (1:3000, 2276, CST); Anti-HA (1:750, 3724, CST); Anti-Tubulin (1:20000, T6199, Sigma-Aldrich); Anti-Drosha (1:2000, 3364, CST; 1,2000, SC393591, Santa Cruz Biotechnology; 1:2000, ab12286, abcam); Anti-Dnmt1 (1:2000, 5032, CST); Anti-Dnmt3A (1:3000, SC20703, Santa Cruz Biotechnology); Anti-Dnmt3B (1:3000, SC52922, Santa Cruz Biotechnology); Anti-Dnmt3L (1:1000, 12309, CST); Anti-Uhrf1 (1:800, SC98817, Santa Cruz Biotechnology); Anti-Histone H3 (1:2000, ab1791, abcam); Anti-Tet1 (1:1000, 09–872, Millipore); Anti-Tet2 (1:1000, ABE364, Millipore); Donkey anti-rabbit HRP (1:5000, NA934V, GE Healthcare); Sheep anti-mouse HRP (1:5000, NA931V, GE Healthcare).

### MethylC-seq library preparation, sequencing and methylation level calculation

Libraries, analysis of sequencing data and calculation of weighted methylation levels were as described in Vlachogiannis *et al.* (16).

### RNA-seq library preparation

Total RNA was purified from ES cells using TRIzol Reagent (ThermoFisher Scientific), before treating with DNase Turbo (Ambion). RNA was then purified into mRNA and short and long RNA using Oligotext mRNA Mini Kit (Qiagen) and *mir*Vana™ miRNA Isolation Kit (ThermoFisher Scientific), respectively. ERCC RNA Spike-In Mix (ThermoFisher Scientific) was added to all RNA samples. Post-purification, short RNAs were treated with Ribo-Zero (Illumina) to remove rRNA. Post purification, mRNAs were fragmented by magnesium treatment followed by phosphatase-mediated repair of cleaved ends using Antarctic Phosphatase (NEB) and T4 Polynucleotide Kinase (PNK) (NEB). Following a final cleanup using RNeasy Kit (Qiagen), sequencing libraries were generated for both mRNA and short RNA fractions using NEBNext Multiple Small RNA Library Prep Protocol (NEB). Depending on the starting amount of input RNA, between 10 and 12 cycles were used for amplification, followed by size fractionation by gel electrophoresis. Amplicons in the 130–350 bp size range were gel-excised and purified using Mini-Elute Gel Extraction Kit (Qiagen), followed by real-time PCR (qPCR)-based quantification using Kapa Library Quantification Kit (Kapa Biosystems) and analysis of size distribution using a Bioanalyzer High Sensitivity DNA Chip (Agilent). Samples were sequenced on a NextSeq 500 (Illumina).

### Expression analysis of RNA-seq data

Adapter sequence was removed from fastq reads using fastq-mcf (−S, −t 0.0001, −l 20). Reads were then trimmed according to quality score using seqtk trimfq (−q 0.01) and aligned to the mouse genome (mm10) with TopHat2 (v2.0.9, –b2-very-sensitive, −g 2, −p 4, –library-type fr-secondstrand, default settings for –mate-inner-dist and –mate-std-dev). Strand-specific bigWig files for visualization of the data in the UCSC Genome Browser were generated using only TopHat2 mapped reads with unique hits on the genome using a custom pipeline incorporating Samtools to select for strand-specific reads, the bedtools genomecov tool and the bedGraphToBigWig tool from UCSC. Transcripts were assembled for wild-type and *Drosha^−/−^* datasets respectively for each of the RNA types (short, long and mRNA) using Cufflinks v2.1.1, with default settings (−g gencode.vM3.annotation.gtf). Wild-type transcripts were then merged with *Drosha^−/−^* transcripts to obtain master transcriptomes for each pairwise comparison of gene expression, in each RNA type, allowing differential gene expression to be assessed using cuffdiff. Differential expression analysis of *Dnmt3* isoforms in the mRNA dataset was performed as part of the Cufflinks pipeline, prior to differential gene expression analysis.

### Accession number

The data generated for this study have been deposited in the Gene Expression Omnibus (http://www.ncbi.nlm.nih.gov/geo/) and are accessible through accession number GSE86907.

### Design of gRNA and CRISPR/Cas targeting

A suitable target of CRISPR sgRNA within the *Drosha* locus was identified according to the rules outlined in Mali *et al.* (17). Briefly, the selected target sequence (5′-GCA CCG AGA TCA CAG TCA C-3′) was incorporated into a 455 bp fragment containing all the necessary components for guide RNA (gRNA) expression (U6 promoter, gRNA scaffold and termination signal) and synthesized as a gene block (Integrated DNA Technologies, IDT). This gRNA fragment was polymerase chain reaction (PCR) amplified prior to use for transfection. In addition, a *neo*-replacement cassette with 75 bp homology arms either side of the Cas9 cut site was synthesized by PCR amplification and included for homology-dependent repair. On the day of transfection, 5 × 10^6^ ES cells were seeded onto a 6 cm tissue culture plate and allowed to attach for at least six hours. Cells were transfected with PCR amplified gRNA, *neo*-replacement cassette and hCas9 expressing plasmid (hCas9, 41815, Addgene) using Effectene transfection reagent (Qiagen) according to the manufacturer’s protocol. A total of 15 μg of DNA was used per transfection. Cells were transfected for up to 20 h, before re-plating at low density on gelatinized 15 cm tissue culture plates. Twenty-four hours after seeding, cells were placed under selection by the addition of G418 (300 μg/ml) to the culture media. After 6–10 days, individual colonies were picked and expanded. For additional *Drosha*-deficient clones, the following modifications were made. The *neo*-replacement cassette was omitted and transfected DNA included a puromycin-resistance plasmid (pSUPER.retro.puro, Oligoengine). Twenty-four hours after seeding, cells were placed under puromycin selection for 48 h before replacing with and further culturing in drug-free ES cell media.

### Screening for *Drosha*-deficiency

PCR primers outside of the homology arms of the replacement cassette were designed and used to detect clones that had undergone homology-dependent repair and insertion of the selection cassette. 5′ and 3′ junctions were analysed by PCR followed by Sanger sequencing. Candidate clones were analysed by western blot using Drosha antibody to confirm absence of the protein.

### Generation of DROSHA expression construct

Mouse *Drosha* (AK148640/7120429A12) complementary DNA (cDNA) was obtained from Source BioScience. PCR primers were designed to amplify up full-length mouse *Drosha* before cloning into the multiple cloning site of pEGFP-C1 expression vector (Clonetech) and pcDNA3-HA (ThermoFisher Scientific). Fragments corresponding to different regions of mouse DROSHA were designed and generated by PCR using full-length *Drosha* cDNA according to previously published human fragments (18). For fragments 2, 3 and 4 that lack the endogenous DROSHA NLS, the SV40 large T antigen NLS (PKKKRKVEDP) was inserted in-frame and downstream of the HA epitope in the pcDNA3-HA cloning vector. For DROSHA E1045Q mutation, site-directed mutagenesis was performed on pcDNA3-HA containing wild-type full-length *Drosha* cDNA using Q5^®^ Site Directed Mutagenesis Kit (NEB). Sanger sequencing verified that all constructs were of correct sequence.

### Immunoprecipitation

For FLAG immunoprecipitation of protein from *Dnmt1^Tag/+^* cells, two methods were used. For mass spectrometry analysis, harvested cells were washed twice in cold 1× Dulbecco's PBS before re-suspending in a hypotonic buffer (10 mM 4-(2-hydroxyethyl)-1-piperazineethanesulfonic acid (HEPES), pH 7.65, 10 mM MgCl_2_, 10 mM KCl, 0.5 mM Dithiothreitol (DTT), 1× ethylenediaminetetraacetic acid (EDTA)-free Protease Inhibitor (Roche)). Cells were lysed by Dounce homogenization. The cytosolic fraction was separated by centrifugation at 4°C (228 rcf, 10 min). The remaining nuclear fraction was washed three times with Buffer N (15 mM HEPES, pH 7.65, 10 mM MgCl_2_, 0.5 mM DTT, 250 mM sucrose, 1× EDTA-free Protease Inhibitor). Nuclei were then re-suspended in a high salt buffer (5 mM HEPES, pH 7.65, 25% glycerol, 10 mM MgCl_2_, 0.2 mM EDTA, 0.5 mM DTT, 250 mM NaCl, 1× EDTA-free Protease Inhibitor), with the NaCl concentration increased to 300 mM by drop-wise addition of 5 M NaCl. Nuclei were lysed in the presence of 75U of Universal Nuclease (Pierce) by incubation at 4°C for 2 h with agitation before centrifugation at 4°C (12 000 rcf, 20 min) to separate out the soluble and insoluble/chromatin nuclear fractions. FLAG-tagged Dnmt1 protein was immunoprecipitated using anti-FLAG^®^ M2 Magnetic Beads (Sigma-Aldrich) according to the manufacturer’s protocol and eluted with FLAG peptide (100 ng/ml, Sigma-Aldrich).

For co-immunoprecipitation followed by western blot, ES cells lysates were prepared by harvesting cells and lysing at a concentration of 2 × 10^7^ cells/ml in Lysis Wash Buffer (20 mM Tris–HCl, pH 7.4, 200 mM NaCl, 2.5 mM MgCl_2_, 0.05% Tween-20) supplemented with Protease Inhibitor Cocktail (Roche). Cells were lysed using a microtipped Misonix Sonicator 3000 (Misonix) (Output 1, 9 pulses for 15 s with 10-s recovery periods). Post-sonication, samples were clarified by centrifugation (10 000 rcf, 10 min, 4°C) before incubating with anti-FLAG^®^ M2 Magnetic Beads as described above. Post-immunoprecipitation, beads were washed and bound material released by incubation in SDS-Page Sample Buffer at 95°C for 10 min. Samples were electrophoresed on 4–15% gradient SDS-PAGE acrylamide gel.

### Immunofluorescence

COS-7 or NIH/3T3 cells were grown on coverslips and co-transfected with DsRed-DNMT1 (human) (19) and HA-DROSHA (mouse) expression constructs using Fugene HD reagent (Promega) according to the manufacturer’s protocol for 48 h. Cells were then fixed with 1% paraformaldehyde. DsRed-DNMT1 was visualized by excitation with 561 nm wavelength, whilst DROSHA detection was by anti-HA antibody (2367, CST) visualized with secondary anti-mouse Alexa Fluor 488 dye (Molecular Probes). Images were acquired on a Zeiss Confocal LSM 880 using Airyscan mode (Zeiss). For quantification of DNMT1 and DROSHA co-localization, Manders split coefficients were determined using the ImageJ JACoP plugin (ImageJ.net). Coefficients represent the number of red spots (DNMT1) coinciding with green spots (DROSHA) with a threshold set at 0.4 for co-localization in individual cells.

### Measurement of genomic 5mC levels by LC-MS/MS

One microgram of genomic DNA was denatured at 98°C for 3 min, incubated on ice for 3 min and digested to single nucleosides using 1 ml of a proprietary blend of nucleases and phosphatases (New England Biolabs) at 37°C overnight. Post digestion, nucleosides were purified using a QIAquick spin column (QIAgen). LC-MS analysis was performed on an Agilent 1200 series HPLC system equipped with a G1316A UV detector and 6120 mass detector (Agilent, Santa Clara, CA, USA) with a Waters Atlantis T3 column (4.6 × 150 mm, 3 μm, Waters (Milford, MA, USA)) equipped with an in-line filter and guard column. Peak quantification was based on the integration area of each target nucleoside at the maximum absorption of ultraviolet (UV) and adjusted by its respective extinction coefficient constant.

### *In vitro* methyltransferase assay

ES cells were lysed and 10 μg of crude cell extract was incubated with 100 ng of hemi-methylated DNA in the presence of 1 μg RNase A for 30 min. Samples were transferred onto filter discs and a liquid scintillation counter used to measure [^3^H]-CH_3_ incorporation into DNA. Two separate measurements were taken for each sample. For human DNMT1 methyltransferase assays in the presence of hairpin RNAs, 10 nM of purified, recombinant DNMT1 was mixed with 10 nM of *in vitro* transcribed and purified RNA and 5 mM of ^3^[H]-SAM and 40 nM of hemi-methylated substrate in methylase buffer.

### *In vitro* transcription of RNA

For *in vitro* human DROSHA processing assays, gBlocks (IDT) corresponding to the hairpins indicated were synthesised with the inclusion of a T7 promoter sequence. gBlocks were used for *in vitro* transcription according to the protocol published by Lee and Kim (20). For *in vitro* transcription followed by incubation with recombinant DNMT1, RNA was synthesized using the same gBlocks and MEGAscript T7 Transcription Kit (Thermo FisherScientific), according to the manufacturer’s protocol.

### *In vitro RNA processing assay by* DROSHA

*In vitro* transcribed RNAs were processed according to the protocol described by Lee and Kim (20).

### Identification of RNA hairpins

Putative hairpins were identified using mFold web server (21).

### Retroviral transduction of *Drosha*-deficient ES cells

Mouse *Drosha* cDNA was cloned into pMSCV-puro (Clontech), sequenced, verified and transfected into Phoenix packaging cells. Briefly, on the day of transfection, 1 × 10^6^ cells were seeded onto 10 cm tissue culture plates and left for 4–5 h to attach. Cells were transfected overnight with 10–14 μg of plasmid using Profection^®^ reagent (Promega), according to the manufacturer’s protocol. The next day, culture medium was replaced with fresh ES cell medium. Forty-eight and seventy-two hours after initial transfection, retroviral particle-containing medium was harvested and syringe-filtered through a 0.45-μ membrane. Virus-containing medium was mixed with polybrene (Sigma-Aldrich) and used to transduce ES cells overnight. ES cells were seeded on the day of transduction (4 × 10^6^ onto a 10 cm tissue culture plate per transduction) and allowed to attach for at least 5 h. The next day, medium was replaced with fresh virus-containing medium and left overnight. The following day, medium was replaced with puromycin-containing ES cell medium (2 μg/ml) and cultured for 6–8 days. Puromycin-resistant colonies were picked and expanded. DROSHA expression was determined by western blot.

### Primers

Sequences of PCR primers used are listed in Supplementary Table S3.

## RESULTS

### Drosha interacts with Dnmt1

**Figure 1.**
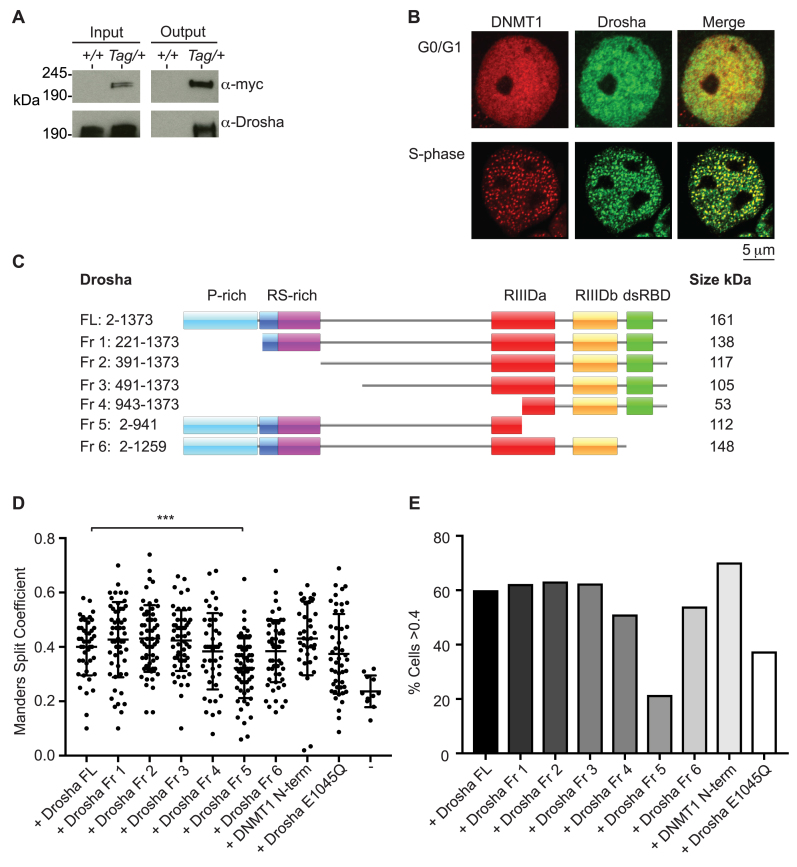
Drosha interacts and co-localizes with Dnmt1. (**A**) Western blot using anti-Drosha antibody following immunoprecipitation with anti-FLAG M2 magnetic beads on material from wild-type (*+/+*) and *Dnmt1^Tag/+^* (*Tag/+*) ES cells. (**B**) Immunofluorescence micrographs of COS-7 monkey kidney cells co-transfected with DsRed-DNMT1 and HA-Drosha constructs. (**C**) Cartoon showing different fragments of Drosha examined and the domains they encompass. (**D**) Scatter plot showing distribution of cells with different Manders Split Coefficient values corresponding to co-localization between DsRed-DNMT1 and the different HA-Drosha constructs indicated. ‘–’ indicates cells transfected with DsRed-DNMT1 only. Dnmt1 N terminal fragment contains amino acids 2–452. Exact Mann–Whitney *P*-values of different fragments compared to distribution using full-length HA-Drosha are Fr1 (0.0201), Fr2 (0.2872), Fr3 (0.3934), Fr4 (0.4197), Fr5 (0.0001), Fr6 (0.3403), DNMT1 N-term (0.1636) and Drosha E1045Q (0.211). (**E**) Bar graph showing percentage of cells analysed with Manders Split Coefficient values of >0.4. Images from at least 50 cells were analysed per construct.

To identify novel factors involved in correct maintenance methylation, we generated ES cells expressing an epitope-tagged version of the maintenance methyltransferase DNMT1 for use in a protein interaction screen. A dual FLAG-c-myc tag was introduced after the start methionine by homologous recombination in ES cells (Supplementary Figure S1A). Following confirmation of correct targeting at the endogenous locus (Supplementary Figure S1B), and Cre-mediated removal of the floxed *neo*-selection cassette, western blot using both anti-FLAG and anti-c-myc antibodies confirmed the presence of signal at the expected size in the targeted cell lines only (Supplementary Figure S1C). This cell line is hereafter referred to as *Dnmt1^Tag/+^*. The FLAG-epitope was subsequently used for native immunoprecipitation of tagged bait and associating proteins. Nano-LC-MS/MS analysis of these interacting proteins isolated from *Dnmt1^Tag/+^* cells conclusively identified the Class 2 RNase III enzyme DROSHA (Supplementary Figure S2A and Table 1). In addition, we detected peptides that identified proteins corresponding to known DNMT1 and DROSHA interactors (UHRF11 and PCNA and DGCR8, respectively). Together with the double stranded RNA-binding protein DGCR8, DROSHA forms Microprocessor complex, which processes long primary miRNAs (pri-miRNAs) into short hairpin precursor miRNAs (pre-miRNAs). As well as this canonical function in miRNA biogenesis, DROSHA has also been shown to process longer RNA species that contain stem–loop structures that is necessary for correct, non-miRNA-mediated gene regulation (22–25). We validated DROSHA interaction by western blot using anti-DROSHA antibody on FLAG immunoprecipitated material from *Dnmt1^Tag/+^* cells (Figure 1A). In addition, FLAG immunoprecipitation on material from *Drosha^Tag/Tag^* ES cells followed by western blot revealed co-immunoprecipitation with DNMT1 protein (Supplementary Figure S2B), further validating the interaction. To ensure that the DNMT1–DROSHA interaction was not due to the presence of either FLAG or c-myc epitopes, we performed FLAG-immunoprecipitation using *Uhrf1^Tag/+^* and *Dnmt3B^Tag/+^* ES cells. Drosha was only found to co-immunoprecipitate when purifying for DNMT1 (Supplementary Figure S2C). Interestingly, we were able to co-immunoprecipitate DROSHA with DNMT1 in nuclear extracts prepared from *Dnmt1^Tag/+^* ES cells using an engineered universal nuclease, which digested both DNA and RNA (Supplementary Figure S2D). This suggests that the interaction does not require either nucleic acid to be intact.

**Table 1.** Selected list of peptides identified in anti-­FLAG immunoprecipitated eluate from *Dnmt1Tag/+* ES cells

Protein ID	Molecular weight (kDa)	Exclusive peptide spectrum matches		Unique peptide spectrum matches		Percent Seq. coverage (%)	
		Repeat 1	Repeat 2	Repeat 1	Repeat 2	Repeat 1	Repeat 2
DNMT1	183	586	770	139	160	78	81
UHRF1	88	18	15	14	11	29	19
PCNA	29	0	15	0	8	0	39
DGCR8	86	4	13	4	8	9.2	16
DROSHA	159	7	20	6	13	5.5	12

We next asked whether DNMT1–DROSHA interaction was specific to pluripotent stem cells. Immunofluorescence analyses of COS-7 cells co-transfected with DsRed-DNMT1 (human) and HA-DROSHA (mouse) revealed co-localization of both proteins specifically in S-phase at punctate foci (Figure 1B). The maintenance methylation activity of DNMT1 is known to occur at hemi-methylated DNA following replication. To determine the region(s) of DROSHA required for DNMT1 interaction, we performed co-localization analysis using expression constructs containing different DROSHA domains (Figure 1C and Supplementary Figure S3). Transient transfection in a different cell line (NIH/3T3) again revealed co-localization of DNMT1 and various DROSHA fragments during S-phase (Figure 1D and E; Supplementary Figure S4). However, there was a reduction in the percentage of cells showing co-localization using Fragment 5, which lacks the majority of the RNase catalytic domain, suggesting that the DROSHA C-terminus is required for co-localization with DNMT1. Interestingly, co-transfection of full-length DROSHA with an expression construct encompassing the N-terminus of DNMT1 (amino acids 2–452) showed similar levels of co-localization to that observed using full-length DNMT1 indicating that the N-terminus of DNMT1 is sufficient for co-localization with DROSHA (Figure 1D and E). Co-localization of DROSHA with DNMT1 was not significantly affected in cells transfected with full-length *Drosha* cDNA carrying a point mutation previously shown to disrupt RNA-processing activity (E1045Q mutation). Together, these results demonstrate DROSHA–DNMT1 interaction, that it is not confined to pluripotent stem cells and that it exists in native nuclear extracts under conditions of both normal expression and transient overexpression.

### Generation of *Drosha*-deficient ES cells

**Figure 2.**
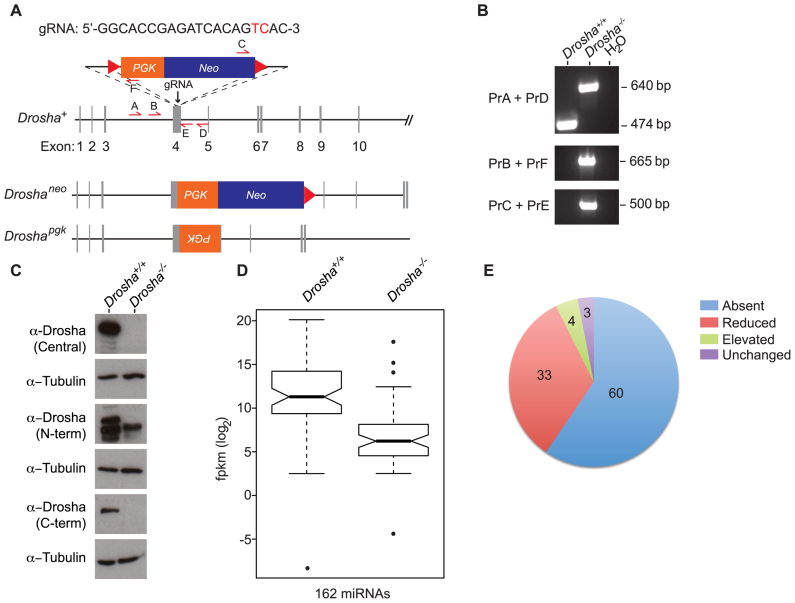
Generation and characterization of *Drosha*-deficient ES cells. (**A**) CRISPR/Cas-mediated gene targeting strategy used to inactivate endogenous Drosha locus. gRNA sequence is shown with hCas9 cut site highlighted in red. PCR primers used for screening are indicated by arrows (A,-E). Alleles generated in *Drosha^−/−^* ES cells following CRISPR/Cas-mediated gene editing. Actual sequence of alleles is shown in Supplementary Figure S3. (**B**) Agarose gel electrophoresis of PCR amplicons used to diagnose targeting of *Drosha* locus. Primers are indicated in (A). (**C**) Western blot using anti-Drosha antibodies recognizing different regions of DROSHA protein on whole cell extracts from wild-type and *Drosha^−/−^* ES cells. Anti-tubulin antibody used as a loading control. Note that the lower band observed using the N-terminal antibody is a non-specific signal. (**D**) Box-whisker plots of RNA-seq data from wild**-**type and *Drosha^−/−^* small RNA libraries. *P* = 4.182 × 10^−10^ (Wilcoxon-rank sum test). Expression levels (log_2_ fragments per kb of exon per million fragments (fpkm)) of 162 miRNAs. (**E**) Pie chart indicating percentage of miRNAs in different categories caused by *Drosha*-deficiency.

The existence of a DNMT1–DROSHA interaction suggested that DROSHA might be required to ensure correct DNA methylation. To examine DROSHA function, CRISPR/Cas technology was used to generate *Drosha*-deficient ES cells (Figure 2A). PCR and sequencing analysis revealed one clone (F10) that carried two different mutations; one mutant allele contained most of the *neo*-replacement cassette whilst the other allele contained part of the *pgk* promoter inserted in inverse orientation (summarized in Figure 2A and B; Supplementary Figure S5). These cells are hereafter referred to as *Drosha^−/−^*. Directed Sanger sequencing also indicated the absence of exonic off-target mutations (Supplementary Figure S6). Western blot data confirmed the absence of detectable full-length DROSHA protein using several antibodies raised against different regions of the protein (Figure 2C). To confirm that these cells were functionally deficient in miRNA biosynthesis, we performed RNA-seq on short RNA libraries generated from *Drosha^−/−^* ES cells. Examination of miRNAs revealed the absence or reduction of 151 (93%) different miRNAs (Figure 2D and E, *P* = 4.182 × 10^−10^, Wilcoxon-rank sum test and Supplementary Table S1). Interestingly, a small percentage of miRNAs is also found to be either unchanged (5%) or elevated (7%) in *Drosha*^−/−^ ES cells. We note that the expression of three of these, *Mir5099, Mir6240* and *Mir320*, have previously been reported to be independent of *Dgcr8* in ES cells, indicating that they are not dependent on Microprocessor activity for their biogenesis (26).

Previous studies on ES cells deficient for the other component of Microprocessor *Dgcr8* indicate that cells unable to generate primiRNAs form smaller colonies in culture and show defects in cell proliferation (27,28). We found that following seeding at the same density and passage duration in culture, *Drosha*-deficient ES cells formed smaller colonies compared to parental wild-type cells (Supplementary Figure S7A). Analysis of RNA-seq data indicated that in general, *Drosha*-deficiency resulted in an increase in markers associated with both naïve and general pluripotency (Supplementary Figure S7B). Cell cycle and viability analysis revealed that as well as reduced proliferation, *Drosha*-deficiency also resulted in increased apoptosis (Supplementary Figure S7C and D). Together, these results confirm previous data indicating that primiRNA processing is necessary for normal ES cell function.

### *Drosha*-deficiency results in reduced cytosine methylation

**Figure 3.**
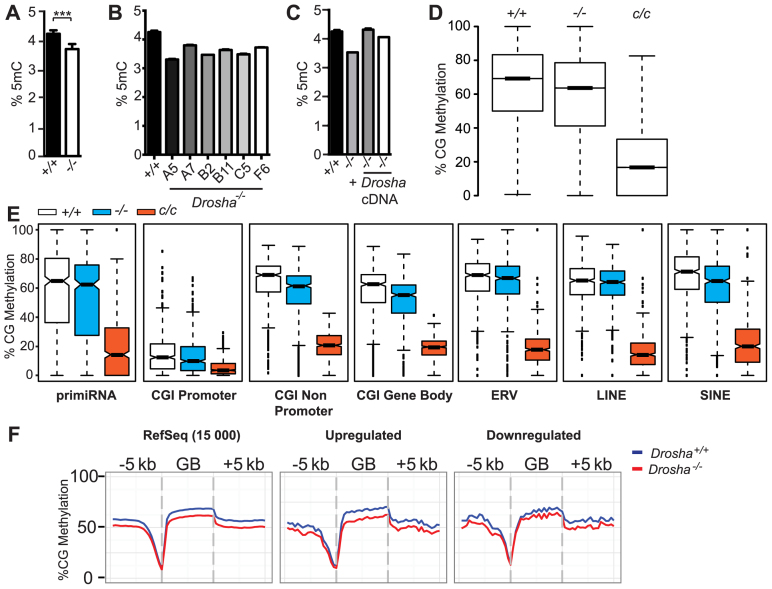
Drosha-deficiency results in global hypomethylation. (**A**) Bar graphs showing LC-MS/MS results measuring percentage of 5mC in genomic DNA extracted from wild-type (*+/+*) and *Drosha*-deficient (−*/−*) ES cells. Exact *P*-value = 0.0007 (Mann–Whitney test). (**B**) Same as (A) but measurements made on genomic DNA extracted from wild-type and various *Drosha*-deficient ES cell clones. (**C**) Same as (A) but measurements made on genomic DNA extracted from wild-type, *Drosha*-deficient ES cells and two independently transduced clones of *Drosha*-deficient ES cells expressing *Drosha* cDNA (+*Drosha* cDNA). (**D**) Box-whisker plots showing the distribution of methylation levels of each cytosine in CG context represented in WGBS data generated from the cell lines indicated. (**E**) Notched box-whisker plots showing weighted CG methylation levels in the various compartments in the different cell lines indicated; *c/c* indicates *Dnmt1^c/c^*, which are deficient for DNMT1 protein. Exact *P*-values (Wilcoxon-rank sum test) for comparisons between wild**-**type and *Drosha*^−/−^ are *P* < 2.2 × 10^−16^. (**F**) Metaplots of CG methylation within gene bodies for 15 000 randomly selected RefSeq genes, genes up- and downregulated in *Drosha^−/−^* compared to wild-type ES cells. Deregulated genes defined as those with a >1.5-fold change and *P*-value < 0.05 in *Drosha*^−/−^ mRNA-Seq dataset compared to wild-type. All bar graphs show mean +/− standard deviation.

We next examined DNA methylation levels in *Drosha^−/−^* ES cells. LC-MS/MS analysis of 5-methyl-cytosine (5mC) levels of genomic DNA indicated this was significantly reduced in *Drosha^−/−^* cells (Figure 3A, *P* = 0.0007), a finding supported by 5mC–DNA ELISA (Supplementary Figure S8A). To exclude the possibility that this observed hypomethylation was due to normal clonal variation, we re-targeted ES cells by CRISPR/Cas using the same gRNA as previously described and generated an additional six independently derived clones deficient for *Drosha*. MiSeq-sequence analysis confirmed mutation at the *Drosha* locus (Supplementary Figure S8B) and western blotting for DROSHA protein confirmed absence of expression (Supplementary Figure S8C). In all cases, genomic 5mC levels were reduced compared to the parental cell line. Methylation levels dropped from 4.36% (129/SvEv) to as low as 3.31% (Clone A5) (Figure 3B). The current mouse reference genome contains 4.37 × 10^7^ mapped CG sites and based on results from our whole genome bisulphite sequencing (WGBS) data (see below), indicating that 64% of cytosines in a CG context are methylated, this implies an absence of methylation at between 157 000 (Clone A7) to over 350 000 CG sites (Clone A5) in *Drosha*-deficient cells. As the mouse reference genome does not include CG-rich tandem repeated sequences such as satellites, this range is likely a conservative estimate. We also tested the direct requirement for DROSHA in regulating correct levels of DNA methylation by using retroviral transduction to re-introduce *Drosha* cDNA into *Drosha^−/−^* cells (Supplementary Figure S8D). This resulted in a recovery in levels of 5mC in two independently transduced clones (Figure 3C). To test the requirement for DROSHA catalytic activity in rescuing genomic hypomethylation, we also transduced *Drosha*-deficient ES cells with *Drosha* cDNA carrying a point mutation previously shown to disrupt RNA-processing activity (E1045Q mutation, (18)). Surprisingly, despite expressing DROSHA protein at lower than endogenous levels observed in wild-type ES cells, these cells actually displayed greater hypomethylation than that observed in the parental, *Drosha*-deficient line (Supplementary Figure S9A and B). This could be due to clonal variation; we note that other independently targeted *Drosha^−/−^* ES cells display a range of hypomethylation compared to wild-type cells (Figure 3B). In ES cells, cytosine methylation occurs in both CG and non-CG contexts, the latter of which is thought to be mediated by *de novo* methyltransferases. Analysis of our WGBS data revealed reductions in non-CG methylation (Supplementary Figure S10A), consistent with the reduction in 5mC observed by LC-MS/MS. Interestingly, we were unable to detect any interaction between Dnmt3 family members and Drosha (Supplementary Figure S10B). These results therefore indicate that Drosha is required to ensure normal levels of genomic cytosine methylation.

### CG methylation is reduced in all genomic compartments in *Drosha*-deficient cells

The reduced methylation observed led us to investigate whether this was a general genome-wide effect or confined to specific genomic compartments. To examine CG methylation at base resolution in an unbiased manner, we performed WGBS on DNA from wild-type, *Drosha^−/−^* and Dnmt1-deficient (*Dnmt1^c/c^*) ES cells. Analysis of the distribution of methylation levels of each cytosine in CG context indicated a reduction in *Drosha^−/−^* cells compared to wild-type (Figure 3D), consistent with our analysis by mass spectrometry. We observed reduced methylation in various sequence compartments analysed (Figure 3E and Supplementary Figure S11A; *P* < 2.2 × 10^−16^ for all compartments). Analysis of a number of different methylation target regions by Bisulphite Amplicon Sequencing (BSAS) recapitulated our WGBS data as well as revealing that re-introduction of Drosha into *Drosha*-deficient ES cells resulted in the recovery of DNA methylation (Supplementary Figure S11B and Table S2). We note that the *Airn/Igf2r* DMR is largely unmethylated in wild-type ES cells and the recovery at this loci in *Drosha*-complemented cells was less than that observed in the other regions analysed. We also examined methylation levels within gene bodies to determine if there was any correlation between gene expression differences observed and the level of CG methylation. Although we observed hypomethylation in gene bodies in *Drosha*-deficient ES cells, this did not correlate with expression. Gene body methylation was reduced to the same degree in both highly and lowly expressed genes, as well at 15 000 randomly sampled RefSeq genes (Figure 3F). We conclude that the hypomethylation observed is genome-wide, consistent with a general defect in the methylation machinery.

### Analysis of components involved in regulating DNA methylation

**Figure 4.**
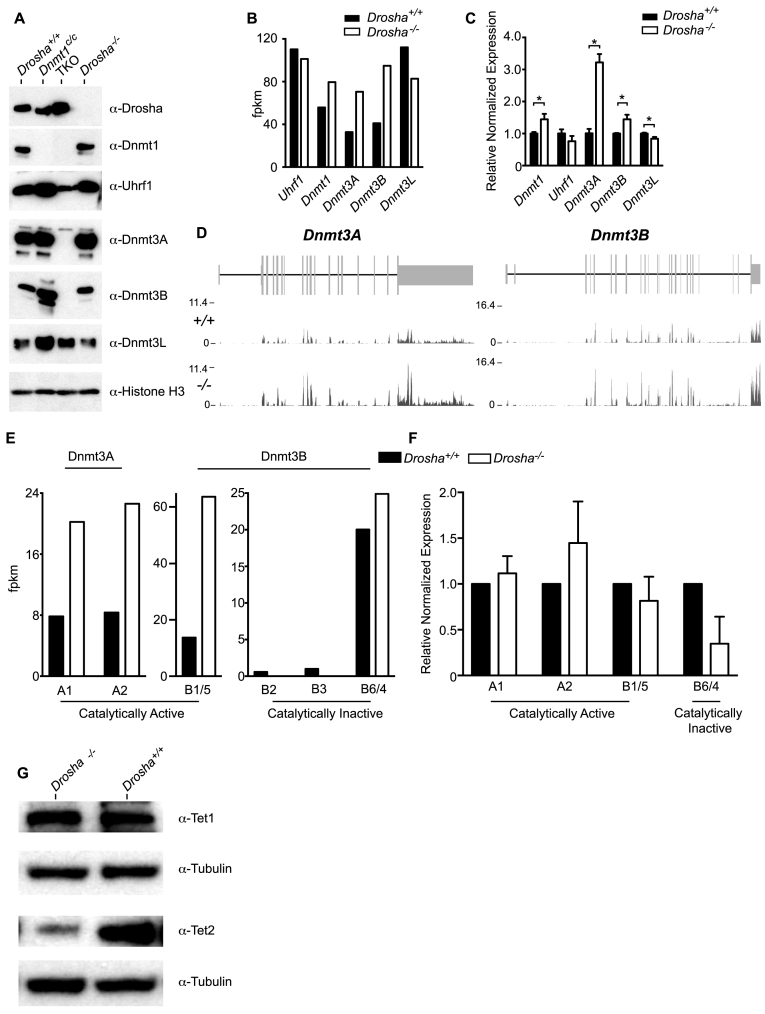
Analysis of components involved in regulating DNA methylation (**A**). Western blot data using the various antibodies indicated on nuclear extracts from the ES cell lines indicated. Triple Knockout (TKO) is an ES cell line deficient for all three active DNA methyltransferases (*Dnmt1^−/−^Dnmt3A^−/−^Dnmt3B^−/−^*). (**B**) Bar graph of RNA-seq data showing expression levels of genes indicated. (**C**) Bar graphs of qRT-PCR data showing expression levels of the factors indicated. Results representative of three biological replicates. For Uhrf1 levels, exact *P*-value = 0.0571 (Mann–Whitney test). For all other genes, *P* = 0.028. *Gapdh* used for normalization. (**D**) RNA-Seq data traces for *Dnmt3A* and *Dnmt3B* loci. (**E**) Bar graphs of RNA-seq data of different *Dnmt3* isoforms indicated. (**F**) Same as (E), but showing Image-J quantified protein levels. (**G**) Western blot data using anti-Tet1 and Tet2 antibody. Anti-tubulin used as loading control.

A number of groups have independently derived *Dicer*-deficient ES cells and these have demonstrated the role of miRNAs in regulating levels of DNMTs. Although not all are in agreement on the effect or precise mechanism, these studies have consistently reported that *Dicer*-deficient ES cells have reduced expression of DNMT3A and DNMT3B (29–31). Analysis of global DNA methylation levels in *Dicer*-deficient ES cells by LC-MS/MS revealed that these are normally methylated, arguing against a general role for miRNA biogenesis in controlling DNA methylation (Supplementary Figure S12). To determine whether the observed hypomethylation was a consequence of reduced expression of DNMTs and known adaptor proteins, we performed western blot analysis. This revealed that steady-state protein levels were unchanged, although there was a trend towards increased expression of DNMT3A and DNMT3B in *Drosha^−/−^* cells (Figure 4A). Analysis of RNA-seq data indicated that *Dnmt1, 3A* and *3B* transcript levels were higher in *Drosha^−/−^* cells, although *Dnmt3L* mRNA levels were reduced (Figure 4B). Analysis of quantitative real-time PCR (qRT-PCR) data revealed that levels of *Dnmt1* and *Dnmt3B* were modestly, but significantly elevated (Figure 4C). In particular, *Dnmt3A* levels showed more than a 3-fold increase in expression whilst levels of *Uhrf1* and *Dnmt3L* were reduced. Together, these data indicate that expression levels of DNMT proteins are their adaptors are unchanged in *Drosha^−/−^* cells.

Recent reports suggest that DNMT3B isoforms lacking catalytic activity are capable of stimulating other catalytic isoforms/partners (32). We therefore considered if the hypomethylation we observed could be attributed to perturbations in the expression of these regulatory isoforms. We re-analysed our mRNA-seq data to determine expression levels of the various known, translated *Dnmt3A* and *Dnmt3B* isoforms (Figure 4D and E). mRNA levels of *Dnmt3A1* and *Dnmt3A2* were both elevated in *Drosha*-deficient ES cells. The predominant *Dnmt3B* isoforms detected were *Dnmt3B1/5* and *Dnmt3B6/4*, which are catalytically active and inactive, respectively. Both isoforms were elevated in *Drosha*-deficient ES cells, although this increase was higher for the catalytically active compared to the inactive isoforms. Quantification of protein levels of different Dnmt3 isoforms recapitulated the RNA-seq data, although we note that levels of the catalytically inactive DNMT3B6/4 isoforms were reduced (Figure 4F). In general, these data suggest that *Drosha*-deficiency results in an increase in all catalytically active DNMT3 isoforms and that alterations in the levels of catalytically active compared to inactive isoforms are unlikely to account for the genomic hypomethylation observed.

TET proteins are capable of oxidizing 5mC to 5hmC and other derivatives and are considered to play a role in both active and passive loss of DNA methylation (33). To examine whether changes in TET expression levels could account for the global hypomethylation observed in *Drosha*-deficient ES cells, we examined the expression levels of both TET1 and TET2, the two predominant Tet enzymes expressed in ES cells by western blot (Figure 4G). This revealed that while TET1 levels are comparable between wild-type and *Drosha*-deficient cells, TET2 levels are actually reduced.

Following methyl extraction by DNMT1, the methyl-donor S-adenosylmethionine (SAM) (used in the methylation of all biological substrates) is converted into S-adenosylhomocysteine (SAH), which has previously been shown to inhibit DNMT activity. To determine whether disturbances in the ratio of SAM and SAH might contribute to the hypomethylation observed, we firstly analysed our RNA-seq data to determine if there were any reductions in the expression of enzymes involved in SAM metabolism (Supplemental Figure S13A). This revealed that while expression of *cystathionine-beta-synthase* (*Cbs*) was reduced in *Drosha*-deficient cells, expression of the other key components involved in SAM metabolism was actually elevated. Furthermore, analysis of the ratio of SAM compared to SAH in cell extracts from Drosha-deficient cells by enzyme-linked immunosorbent assay (ELISA) revealed no significant difference (Supplementary Figure S13B, *P*-value > 0.9999, Mann–Whitney test). These data indicate that defects in SAM–SAH metabolism are unlikely to be responsible for the hypomethylation observed.

Together, these data reveal functional differences between DROSHA and DICER in their regulation of DNMT expression and they suggest that the hypomethylation observed is unlikely to be accounted for by reduced protein levels of the various DNMTs and their adaptors or increased expression of TET1 and TET2.

### DNMT1 methyltransferase activity is reduced by *Drosha*-deficiency

**Figure 5.**
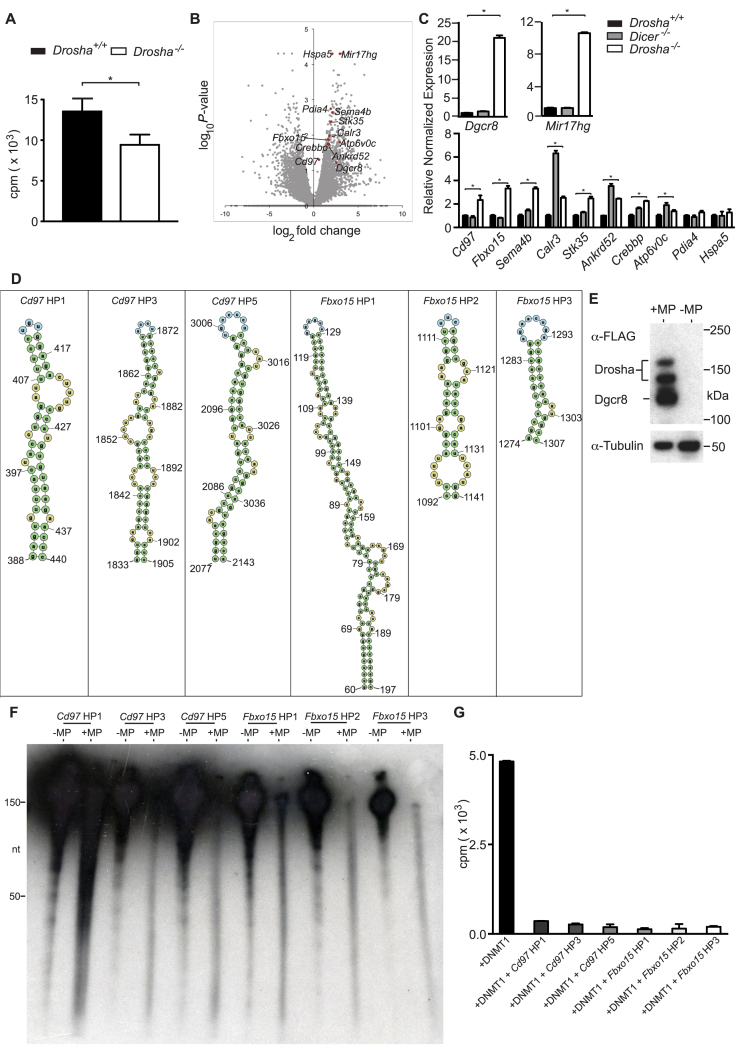
DROSHA upregulated RNAs are processed by Microprocessor and inhibit DNMT1-mediated methyltransferase activity. (**A**) Bar graph showing ^3^H-labelled SAM incorporation into hemi-methylated DNA by lysate from ES cells with the genotypes indicated (*P* = 0.022, Mann–Whitney test). (**B**) Volcano plot of log_2_ fold-change versus log_10_*P*-value of 21 509 RefSeq genes in *Drosha*^−/−^ compared to wild-type ES cells. Previously identified DNMT1 Interacting RNAs (DiRs) not upregulated in *Dicer^−/−^* microarray data highlighted in red, as well as *Mir17hg* and *Dgcr8*. (**C**) Bar graphs showing qRT-PCR expression data of factors highlighted in (B). Results representative of two biological replicates. *P*-values (Mann–Whitney test) are: *Pdia4*, 0.4857 (wild-type versus *Dicer^−/−^*); *Hspa5*, 0.2, >0.99, 0.3429 (wild-type versus *Drosha^−-/−^*, wild-type versus *Dicer^−-/−^, Drosha^−/−^* versus *Dicer^−/−^*, respectively*). P* = 0.028 for all other comparisons. All bar graphs show mean +/− standard deviation. *Gapdh* used for normalization. (**D**) Graphic showing predicted hairpin/stem loop regions within *Cd97* and *Fbxo15* RNA. Numbers indicate base positions. (**E**) Western blot of lysate from HEK293T cells transiently transfected with FLAG-tagged DROSHA and DGCR8 (+MP) probed with anti-FLAG antibody. –MP indicates material from untransfected cells. (**F**) Autoradiograph of polyacrylamide gel-resolved *in vitro* transcribed RNAs indicated incubated with cell extracts from untransfected (–MP) or Microprocessor transfected (+MP) cells. Note the great reduction in signal of unprocessed full-length RNA and appearance of lower molecular weight smear in +MP lanes compared to –MP lanes. (**G**) Bar graph of *in vitro* methyltransferase assays. Cells were transfected with either DNMT1 alone or DNMT1 along with the *in vitro* transcribed RNAs indicated.

In light of our findings that DNMT protein levels were not reduced in *Drosha^−/-^*cells, the genomic hypomethylation observed was unexpected. In addition, we failed to detect any conspicuous perturbation in DNMT1 localization in *Drosha*-deficient ES cells (Supplementary Figure S13), arguing against any gross alterations in DNMT1 sub-cellular localization. We therefore posited whether absence of DROSHA had any effect on DNMT1 activity. Using an *in vitro* methyltransferase assay, we examined whether DNMT1 methyltransferase activity on hemi-methylated DNA, the preferred substrate for this enzyme (34), was altered in *Drosha*-deficient ES cells. Cell extracts from *Drosha^−/−^* ES cells consistently harboured reduced methyltransferase activity compared to extracts from parental wild-type ES cells (Figure 5A, *P* = 0.022, Mann–Whitney test). These data indicate that the presence of Drosha has a stimulatory role on DNMT1-mediated DNA methylation.

### DNMT1-interacting RNAs are upregulated in *Drosha*-deficient cells

Given that the hypomethylation observed in *Drosha*-deficient ES cells occurs without any reduction in the expression of proteins known to impact DNA methylation and that genetically *Drosha* is necessary to stimulate DNMT1 activity, we considered alternative mechanisms to account for our observations. A number of recent studies indicate the ability of RNA to interfere with DNMT1 catalytic activity. In particular, DiRs have recently been reported and proposed to inhibit catalytic activity (14). We therefore wondered if any of the transcripts reported to inhibit DNMT1 activity are also specific targets of DROSHA. To eliminate genes that are targets of miRNA regulation and therefore may not be direct targets for DROSHA, we cross-referenced our transcriptome data from *Drosha*-deficient ES cells with a list of genes shown previously to be upregulated in *Dicer*-deficient ES cells (29) prior to comparing this to a list of 3632 DiRs. This resulted in a list of 10 candidate genes (Figure 5B). qRT-PCR was then used to verify the expression status of these genes with the inclusion of RNA from *Dicer*-deficient ES cells (Figure 5C). As controls, we examined expression of two loci known to generate RNA that is directly cleaved by DROSHA. These were the mRNA *Dgcr8* that generates the other component of Microprocessor complex and the long non-coding RNA (lncRNA) *Mir17hg/miR-17∼92*. Of the genes analysed, two, *Fbxo15* and *Cd97*, were found to be consistently upregulated specifically in *Drosha^−/−^* ES cells. We also observed upregulation of these genes in the other *Drosha*-deficient cell lines generated (Supplementary Figure S15, *P* = 0.0286). These data demonstrate the identification of two transcripts that are specifically upregulated in *Drosha*-deficient ES cells, which have also been previously identified as DiRs.

DROSHA has been shown to process non-miRNA targets at hairpin/stem loop structures (22,35). Using secondary structure prediction software (mFOLD) (21), we identified several putative hairpin regions in *Fbxo15* and *Cd97* (Figure 5D). Using an *in vitro* Microprocessor RNA processing assay, we examined whether RNAs corresponding to these regions could be processed by DROSHA. Cell extracts were prepared from NIH/3T3 cells transiently transfected with FLAG-tagged DROSHA and DGCR8 (Microprocessor, MP) (Figure 5E) before incubation with *in vitro* transcribed RNA corresponding to the different RNAs indicated in Figure 5D. Analysis of the size distribution of RNAs by radiography in the presence of MP+ cell extracts revealed that compared to RNA incubated with MP− cell extract, there was a loss of signal corresponding to full-length hairpins and a concomitant appearance of lower molecular weight RNA for all regions tested, indicative of RNA processing (Figure 5F). Finally, the inclusion of RNAs corresponding to these regions in *in vitro* DNA methyltransferase assays resulted in a substantial reduction in enzymatic activity (Figure 5G). Interestingly, we found that RNA derived from *Pdia4*, a gene unaffected by *Drosha*-deficiency, showed reduced ability to inhibit DNMT1 activity (Supplementary Figure S16). According to RNA secondary structure prediction analysis, the RNAs analysed are not predicted to be capable of forming stem–loop structures. However, the fact that they showed some inhibitory activity on DNMT1 activity suggests the existence of additional mechanisms *in vivo* controlling which RNAs are accessible to both DNMT1 and DROSHA. Together, these data indicate that putative hairpin regions within *Cd97* and *Fbxo15* are capable of being processed by DROSHA and that they can inhibit DNMT1 activity.

## DISCUSSION

Holliday and Riggs originally proposed that the ability to recognize hemi-methylated DNA following semi-conservative replication might be a sufficient mechanism by which DNA methylation is stably inherited (36,37). However, data accumulated since then have revealed the existence of other mechanisms that are necessary to ensure the correct maintenance of methylation patterns. These include the correct targeting of DNMT1 by the chromatin-associated adaptor UHRFf1/NP95 (6,7) and post-translational modifications controlling DNMT1 protein stability (8,9). More pertinent, a recent study revealed that RNAs capable of adopting stem–loop structures are capable of associating with and inhibiting DNMT1 methyltransferase activity (14) and a recent description of the interactome of the lncRNA *Xist* identified DNMT1 as a major interacting factor (38).

Our results indicate that a major RNA-processing enzyme, DROSHA, is also capable of interacting with DNMT1 and that DROSHA is necessary for full methyltransferase activity. It remains unclear if this interaction is direct or indirect. Our IF studies indicate that for efficient co-localization of the proteins to occur, the C-terminal region that contains a substantial portion of the RIIIDa, and all of the RIIIDb and dsRBD is necessary. This obfuscates attempts to determine whether the interaction is needed for DROSHA to stimulate DNMT1 activity as deletion or mutation analysis in this region is likely to have a significant impact on DROSHA RNase activity. Another possibility is that given that both proteins co-localize during S-phase, this interaction may be necessary to help reduce local concentrations of inhibitory RNAs at the replication fork to ensure full maintenance methylation activity.

The genomic hypomethylation that we observed in *Drosha*-deficient ES cells occurs despite increased expression of the active DNA methyltransferases. The role of miRNAs in regulating Dnmt expression levels is controversial. Two studies using the same *Dicer^−/−^* ES cell line reported reduced expression of Dnmt3A and Dnmt3B, due to a loss of expression of the *mir290*-cluster and concomitant upregulation of *Rbl* mRNA, targeted by *mir290* (29,31). In addition, the Benetti study also reported reduced expression of DNMT1. Results from another group using a different *Dicer*-deficient ES cell line concluded the cells had reduced expression of DNMT3A and 3B (30). However, another report using a third, different *Dicer^−/−^* ES cell line reported no consistent difference in DNMT expression or RBL protein expression (39). Our results indicate differences in how DROSHA and DICER control DNMT expression. We propose that a possible explanation for this discrepancy is that DROSHA might act directly on *Dnmt* mRNA.

Recent data by several groups have documented that naïve ES cells (cultured in 2i media) are hypomethylated relative to primed ES cells cultured in Fetal Bovine Serum (FBS) (40–42). One possibility is that the hypomethylation we observe is a consequence of *Drosha*-deficiency resulting in cells being in the naïve state, despite their culture in FBS. We consider this unlikely because (i) cytosine methylation in naïve ES cells is at a substantially lower level than the reduction we observe in *Drosha*-deficient cells and (ii) a hallmark of the naïve state is substantial downregulation of DNMT3 expression, which again is inconsistent with our protein and mRNA expression data.

Our analysis of expression levels of TET1 and TET2 revealed that while the former predominant species is unchanged, TET2 levels are reduced in *Drosha*-deficient ES cells. TET proteins are localized to specific regions of the genome, particularly the promoters of genes associated with pluripotency as well as Polycomb-regulated loci (43,44). As the hypomethylation caused by *Drosha*-deficiency is genome-wide, combined with our analysis of TET1 and TET2 expression this argues that TET-mediated demethylation is unlikely to be involved.

Although most recognized for its role in miRNA biogenesis, DROSHA also has the ability to process other RNAs containing stem–loop structures, such as mRNAs (22–25). DiRs have only recently been reported and more work is required to determine their functional significance (14). We note that DiRs were identified in human promyelocytic cells (HL-60) and it is reasonable to assume that both cell-type and species-specific differences in targets exist. We report the identification of two transcripts, *Fbxo15* and *Cd97*, which are upregulated specifically in *Drosha*-deficient ES cells, which have also been previously reported as DiRs. We find that regions within both transcripts predicted to form stem–loop structures are processed by Microprocessor and that these RNA regions are able to inhibit DNMT1 activity. Whether DROSHA is able to process all DiRs is unknown. Based on our genetic data indicating the involvement of *Drosha* in regulating normal genomic levels of DNA methylation and that DROSHA is necessary for full DNMT1 activity, we propose that DROSHA is required for the correct processing of inhibitory DiRs, thereby allowing DNMT1 to achieve full methyltransferase activity.

## ACCESSION NUMBER

The data generated for this study have been deposited in the Gene Expression Omnibus (http://www.ncbi.nlm.nih.gov/geo/) and are accessible through accession number GSE86907.

## Supplementary Material

Supplementary Data
