# A novel requirement for DROSHA in maintenance of mammalian CG methylation

**DOI:** 10.1093/nar/gkx736

**Published:** 2017-08-17

**Authors:** Athanasia Stathopoulou, Jyoti B. Chhetri, John C. Ambrose, Pierre-Olivier Estève, Lexiang Ji, Hediye Erdjument-Bromage, Guoqiang Zhang, Thomas A. Neubert, Sriharsa Pradhan, Javier Herrero, Robert J. Schmitz, Steen K.T. Ooi

**Affiliations:** 1 Department of Cancer Biology, UCL Cancer Institute, London WC1E 6BT, UK; 2 Bill Lyons Informatics Centre, UCL Cancer Institute, London WC1E 6BT, UK; 3 New England Biolabs, 240 Country Road, Ipswich, MA 01938, USA; 4 Institute of Bioinformatics, University of Georgia, 120 East Green Street, Athens, GA 30602, USA; 5 Department of Biochemistry and Molecular Pharmacology, Skirball Institute, NYU School of Medicine, New York, NY 10016, USA; 6 Department of Genetics, University of Georgia, 120 East Green Street, Athens, GA 30602, USA

*Nucleic Acids Res* (2017). doi: 10.1093/nar/gkx695

The name of Hediye Erdjument-Bromage was misspelled in the original version of this paper. This has now been corrected online and in print.

