# Supplementary material for: A novel requirement for DROSHA in maintenance of mammalian CG methylation
Source: Nucleic Acids Res. 2017 Aug 3;45(16):9398–412. doi: 10.1093/nar/gkx695 (PMC5766157; doi:10.1093/nar/gkx695)
Supplement: Supplementary Data [file gkx695_supp.pdf]

Supplementary Information

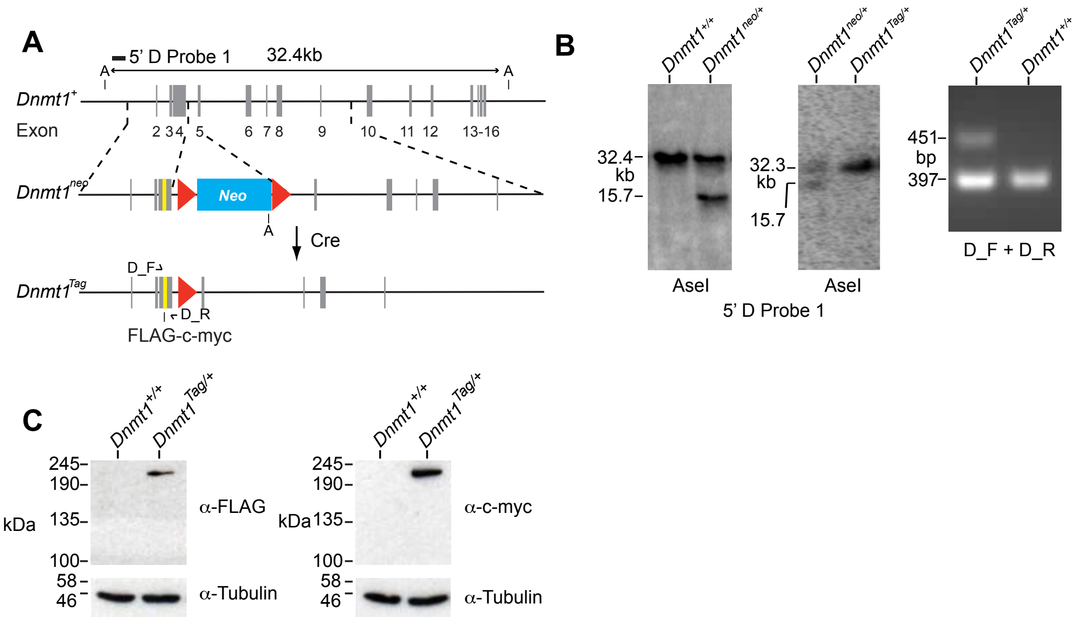

Supplementary Figure S1. Generation of epitope-tagged Dnmt1. (**A**)**.** Targeting strategy used to introduce and detect a dual FLAG-c-myc epitope tag into the endogenous *Dnmt1* locus. Identification of targeted clones involved digestion with AseI (A), restriction endonuclease. (**B**)**.** Diagnostic Southern blot using the probe indicated in (**A**) showing detection of targeted alleles and agarose gel electrophoresis of PCR amplicons used to distinguish between wild type and targeted alleles. (**C**) Western blot data using either anti-FLAG or anti-c-myc antibodies. Bands are as expected based on predicted protein sizes. Note the absence of signal in the untargeted parental cell lines.

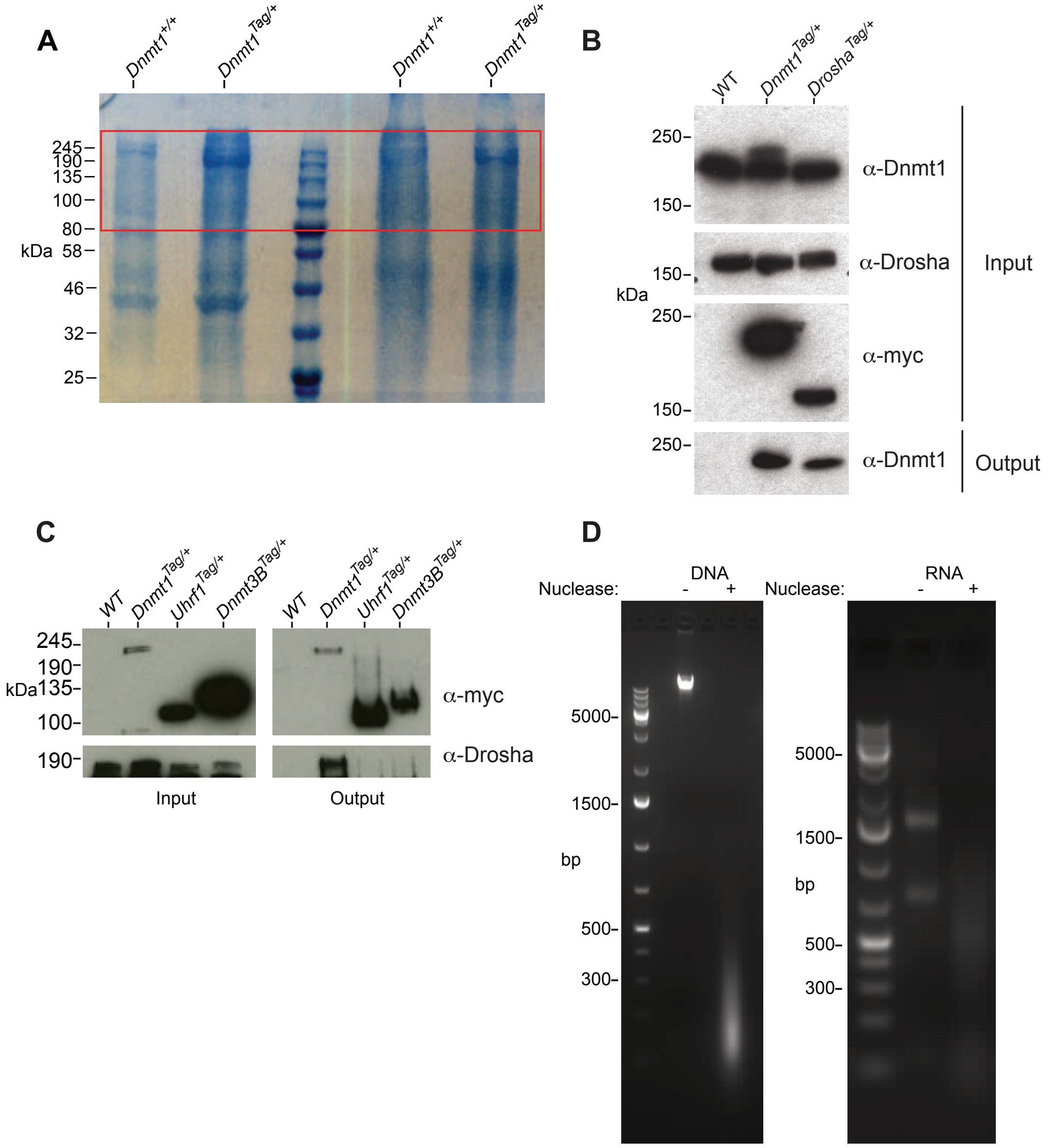

Supplementary Figure S2. Identification of Drosha as a Dnmt1 interacting factor. (**A**) Image of polyacrylamide-resolved material following immunoprecipitation using anti-FLAG antibody using either *wild type* or *Dnmt1*$^{Tag/+}$ ES cell lysate. Red box indicates region excised and subjected to mass spectrometry analysis. (**B**) Western blot data of protein extracts before and after immunoprecipitation with anti-FLAG antibody using material from cell lines indicated. *Drosha*$^{Tag/Tag}$ cells are homozygous for a dual FLAG-myc epitope introduced into the N-terminus of the endogenous Drosha locus in mouse ES cells. (**C**) Same as (**B**) using material from the cell lines indicated. Both *Uhrf1*$^{Tag/+}$ and *Dnmt3B*$^{Tag/+}$ ES cells have a dual FLAG-myc epitope introduced into the endogenous Uhrf1 and Dnmt3B locus, respectively. Note, *WT* and *Dnmt1*$^{Tag/+}$ data are the same as shown in Figure 1A. (**D**) Agarose gels showing DNA and RNA integrity following cell lysis and purification after digestion with Universal Nuclease (Pierce).

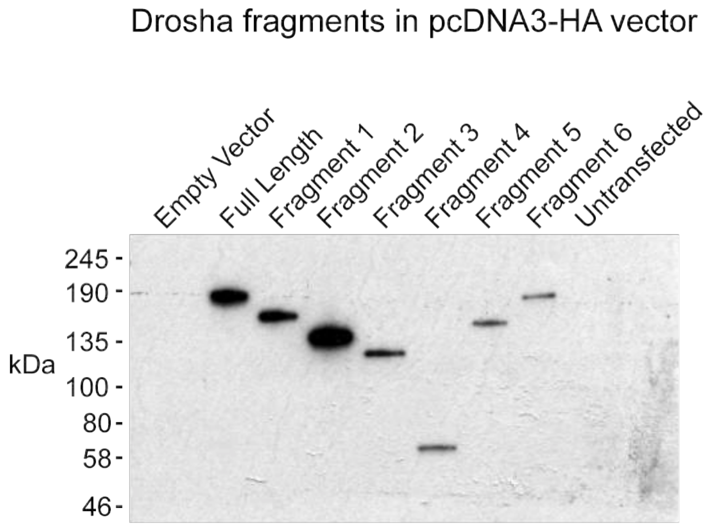

Supplementary Figure S3. Expression of Drosha fragments.

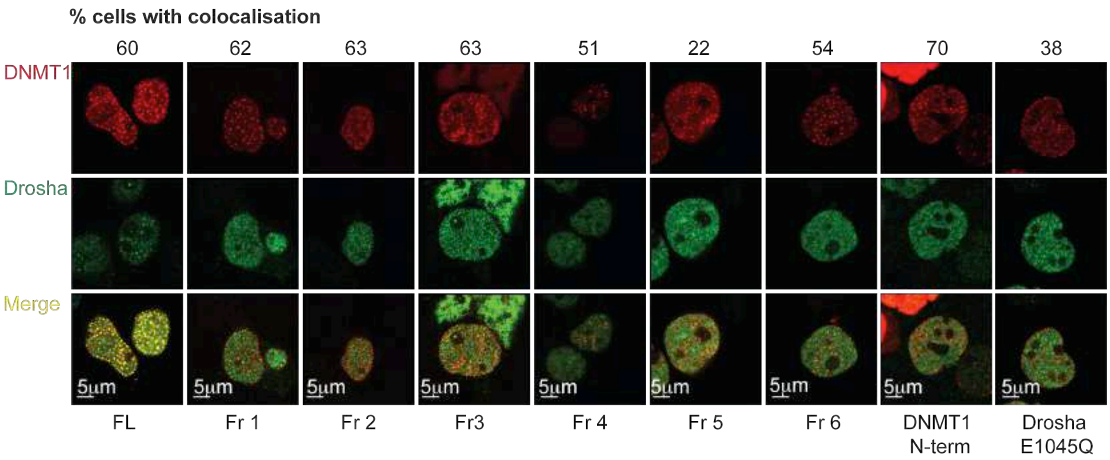

Supplementary Figure S4. Co-localisation of transfected DNMT1 and Drosha. Immunofluorescence micrographs of NIH/3T3 cells co-transfected with DsRed-DNMT1 and HA-Drosha constructs. Numbers indicate the percentage of cells showing signs of signal co-localisation (threshold value 0.4).

Drosha ^{pgk}

5′-GGCACCGAGATCACAGTGAGGCCCTTCGGGCGCCCGCCCCGCCCCTGAGCCCGC
CCCTGAGCCCGCCCCCGGACCCACCCCTTCCCAGCCTCTGAGCCCAGAAAGCGAAGG
AGCAAAGCTGCTATTGGCCGCTGCCCCAAAGGCCTACCCGCTTCCATTGCTCAGCGG
TGCTGTCCATCTGCACGAGACTAGTGAGACGTGCTACTTCCATTTGTCGCGTCGTGC
CACGGGCGAG-3′

__: *pgk* promoter

Drosha ^{neo}

5′-GGCACCGAGATCATACGAAGTTATTAGGTCTGAAGAGGAGTTTACGTCCAGCCA
AGCTAGCTTGGCTGCAGGTCGTCGAAATTCTACCGGGTAGGGGAGGCGCTTTTCCCA
AGGCAGTCTGGAGCATGCGCTTTAGCAGCCCCGCTGGGCACTTGGCGCTACACAAGT
GGCCTCTGGCCTCGCACACATTCCACATCCACCGGTAGGCGCCAACCGGCTCCGTTC
TTTGGTGGCCCCTTCGCGCCACCTTCTACTCCTCCCCTAGTCAGGAAGTTCCCCCCC
GCCCCGCAGCTCGCGTCGTGCAGGACGTGACAAATGGAAGTAGCACGTCTCACTAGT
CTCGTGCAGATGGACAGCACCGCTGAGCAATGGAAGCGGGTAGGCCTTTGGGGCAGC
GGCCAATAGCAGCTTTGCTCCTTCGCTTTCTGGGCTCAGAGGCTGGGAAGGGGTGGG
TCCGGGGGCGGGCTCAGGGGCGGGCTCAGGGGCGGGGCGGGCGCCCGAAGGTCCTCC
GGAGGCCCGGCATTCTGCACGCTTCAAAAGCGCACGTCTGCCGCGCTGTTCTCCTCT
TCCTCATCTCCGGGCCTTTCGACCTGCAGCCTGTTGACAATTAATCATCGGCATAGT
ATATCGGCATAGTATAATACGACAAGGTGAGGAACTAAACCATGGGATCGGCCATTG
AACAAGATGGATTGCACGCAGGTTCTCCGGCCGCTTGGGTGGAGAGGCTATTCGGCT
ATGACTGGGCACAACAGACAATCGGCTGCTCTGATGCCGCCGTGTTCCGGCTGTCAG
CGCAGGGGCGCCCGGTTCTTTTTGTCAAGACCGACCTGTCCGGTGCCCTGAATGAAC
TGCAGGACGAGGCAGCGCGGCTATCGTGGCTGGCCACGACGGGCGTTCCTTGCGCAG
CTGTGCTCGACGTTGTCACTGAAGCGGGAAGGGACTGGCTGCTATTGGGCGAAGTGC
CGGGGCAGGATCTCCTGTCATCTCACCTTGCTCCTGCCGAGAAAGTATCCATCATGG
CTGATGCAATGCGGCGGCTGCATACGCTTGATCCGGCTACCTGCCCATTCGACCACC
AAGCGAAACATCGCATCGAGCGAGCACGTACTCGGATGGAAGCCGGTCTTGTCGATC
AGGATGATCTGGACGAAGAGCATCAGGGGCTCGCGCCAGCCGAACTGTTCGCCAGGC
TCAAGGCGCGCATGCCCGACGGCGATGATCTCGTCGTGACCCATGGCGATGCCTGCT
TGCCGAATATCATGGTGGAAAATGGCCGCTTTTCTGGATTCATCGACTGTGGCCGGC
TGGGTGTGGCGGACCGCTATCAGGACATAGCGTTGGCTACCCGTGATATTGCTGAAG
AGCTTGGCGGCGAATGGGCTGACCGCTTCCTCGTGCTTTACGGTATCGCCGCTCCCG
ATTCGCAGCGCATCGCCTTCTATCGCCTTCTTGACGAGTTCTTCTGAGGGGATCAAT
TCTCTAGAGCTCGCTGATCAGCCTCGACTGTGCCTTCTAGTTGCCAGCCATCTGTTG
TTTGCCCCTCCCCCGTGCCTTCCTTGACCCTGGAAGGTGCCACTCCCACTGTCCTTT
CCTAATAAAATGAGGAAATTGCATCGCATTGTCTGAGTAGGTGTCATTCTATTCTGG
GGGGTGGGGTGGGGCAGGACAGCAAGGGGGAGGATTGGGAAGACAATAGCAGGCATG
CTGGGGATGCGGTGGGCTCTATGGCTTCTGAGGCGGAAAGAACCAGCTGGGGCTCGA
CTAGAGCTTGCGGAACCCTTAATATAACTTCGTATAATGTATGCTATACGAAGTTAT

: *lox*P
: *pgk* promoter
: *neo*

Supplementary Figure S5. Sequences of different CRISPR/Cas-induced mutant *Drosha* alleles.

Gene	Sequence	No. Mismatches with gRNA
Amh	GGCCCCCAGGTCACAGTCAC AGG	4
Rapgef6	GGCACAGGGCTCACAGTCAG TGG	4
Lamb1	GCCCCTGGGATCACAGTCAC AGG	4
Pkhd1l1	GGCACCAGGCTTACAGTCAC AGG	4
Amhr2	GGCCCAGAGCTCACAGGCAC TGG	4
Six2	AGCACGGAGGTCACAGTCCC AGG	4
Pear1	GGCGCCAGGAGCACAGTCAC AGG	4
Asphd2	TGCTCCGTGATCACAGTCCC AGG	4
Vsig10l	GGCAACCAGATCACCCTCAC AGG	4
Ankrd42	GGCGCGGAGACCACAGTCTC CGG	4
Cx3cl1	TGCACCAAGGTGACAGTCAC AGG	4

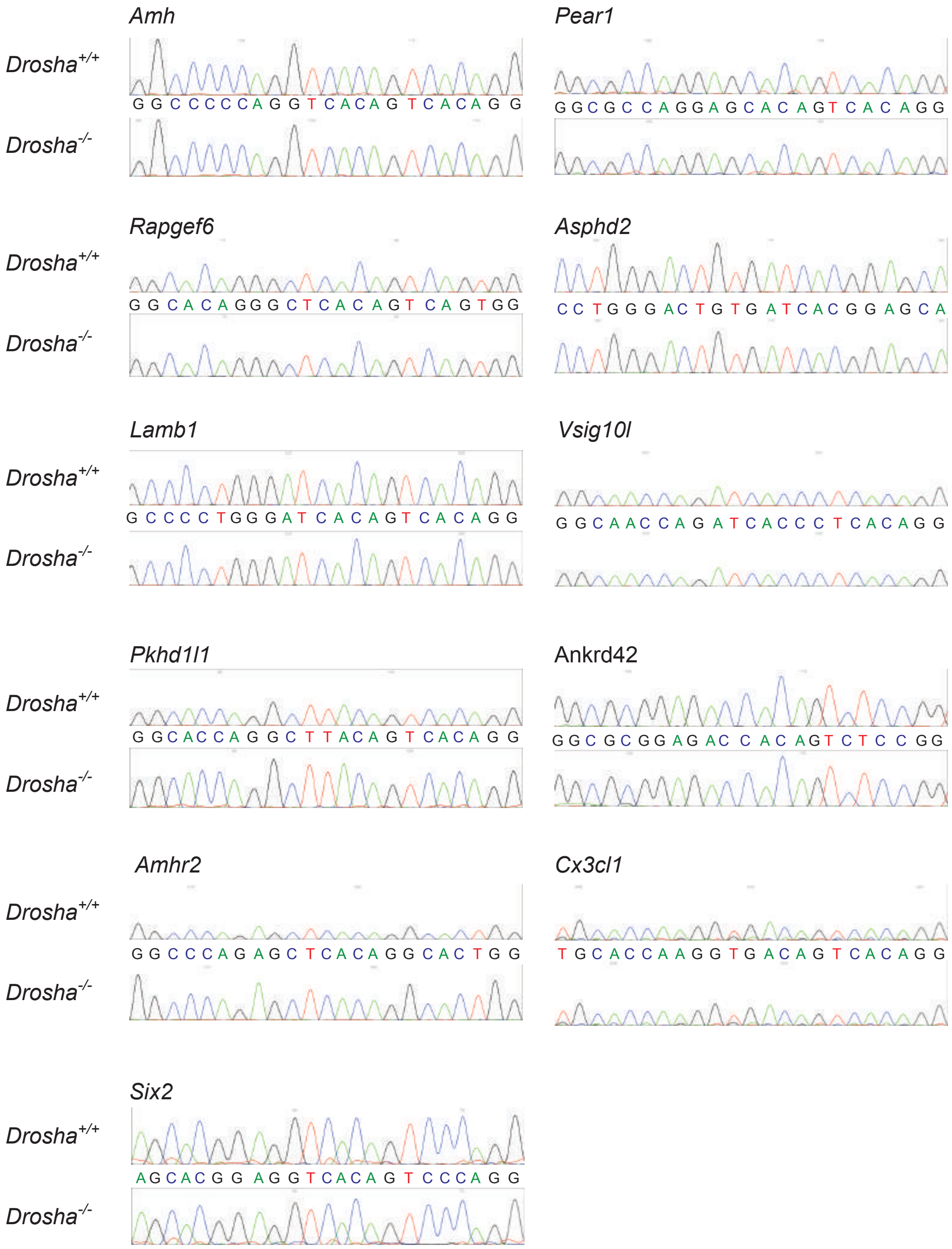

Supplementary Figure S6. Sequence analysis of potential off-target sites with partial homology to gRNA sequence used to edit *Drosha* locus.

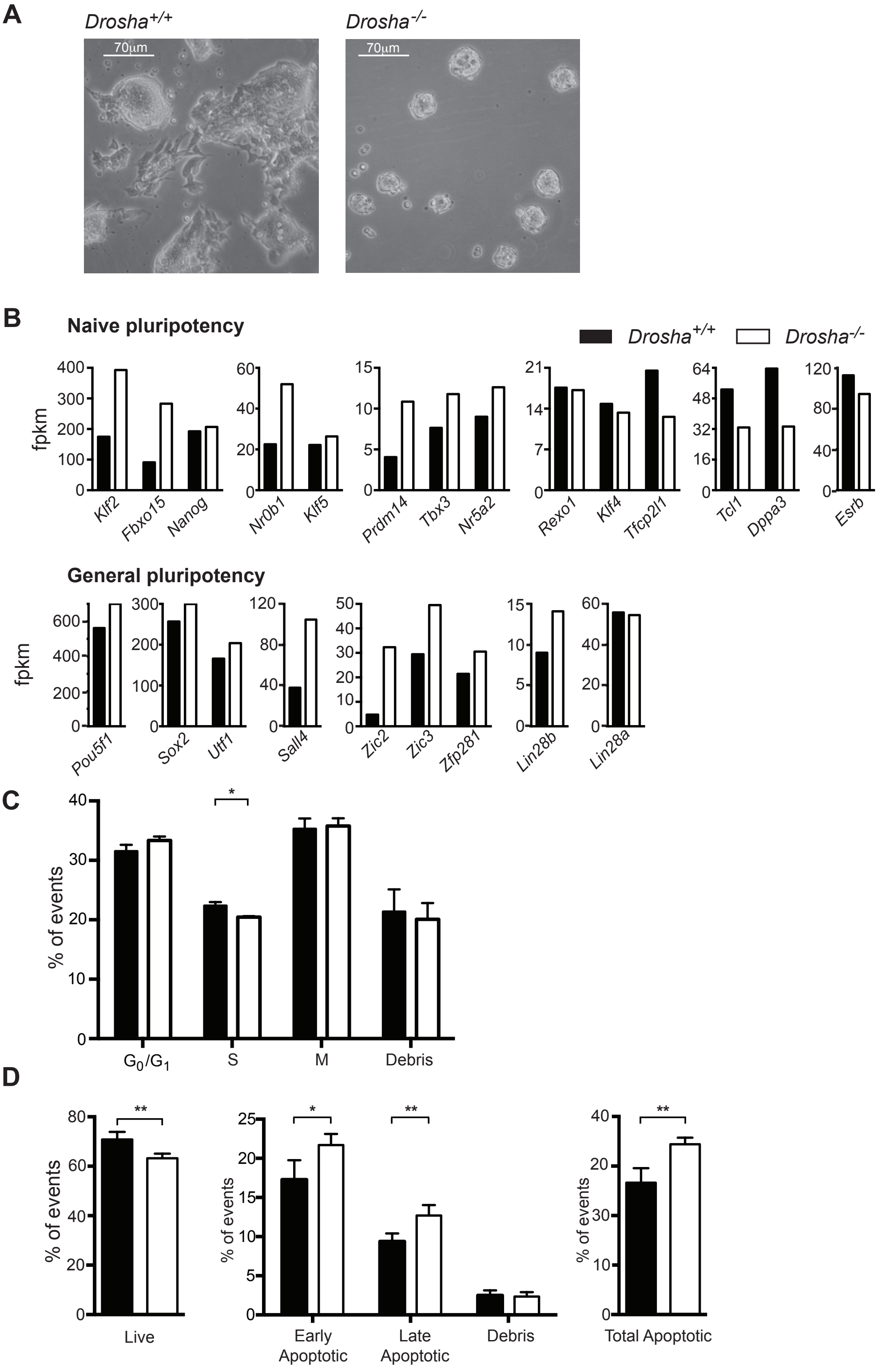

Supplementary Figure S7. Characterisation of *Drosha*$^{-/-}$ ES cells. (**A**) Representative micrographs of ES cell colonies of the genotypes indicated, 3 days post-seeding. (**B**) Bar graphs of RNA-seq data showing expression of indicated markers of pluripotency. (**C**) Bar graphs showing the proportion of events in the different stages of the cell cycle indicated, 48 hours post initial seeding. S-phase exact p-value = 0.0286 (Mann Whitney test). (**D**) Bar graphs showing viability distribution of wildtype and Drosha-deficient ES cells, 48 hours post-initial seeding. Exact p-values (Mann Whitney test): 0.0043 (Live), 0.0173 (Early Apoptosis), 0.0043 (Late Apoptosis), 0.0043 (Total Apoptosis)

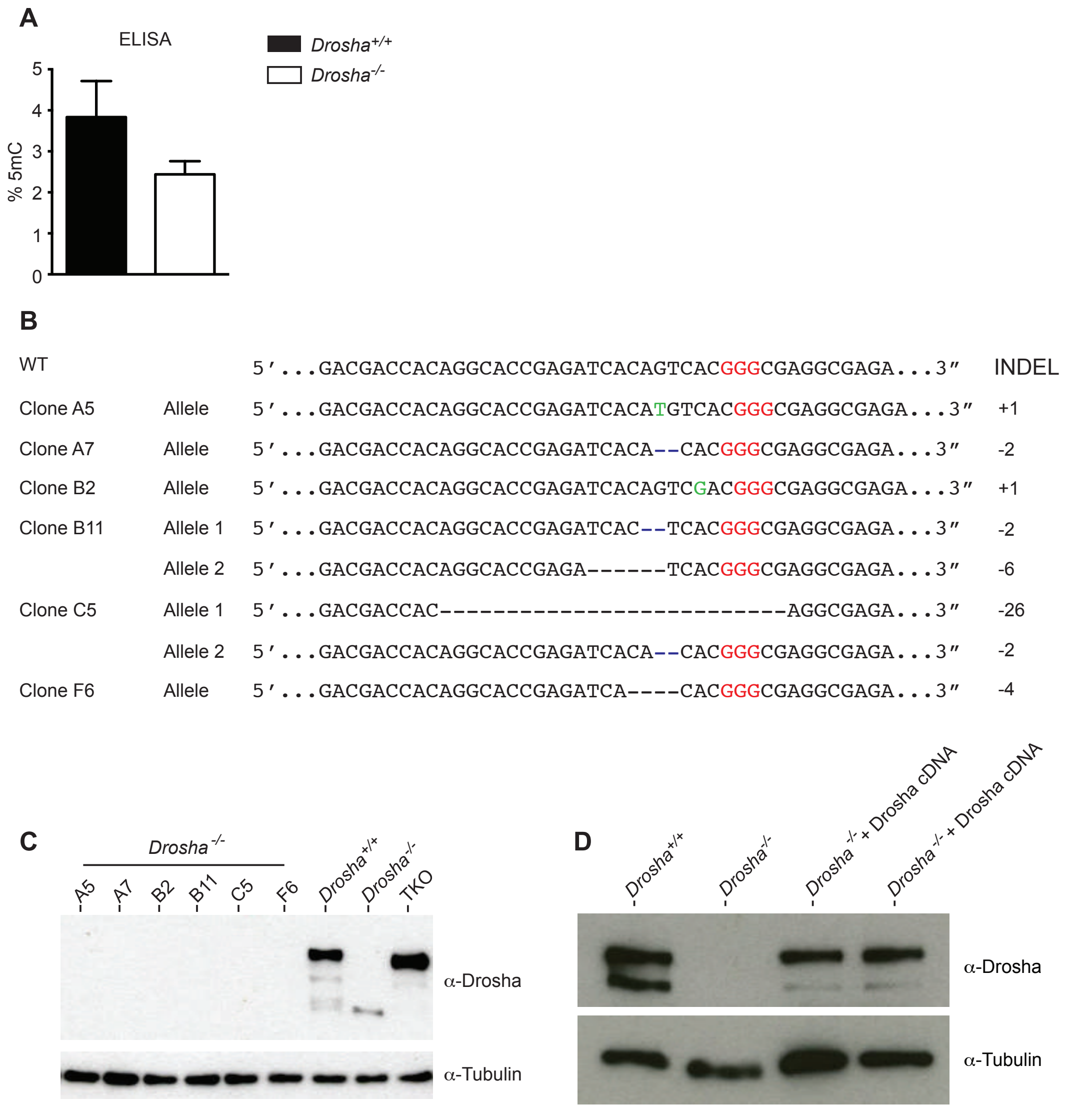

Supplementary Figure S8. Cytosine methylation analysis by 5mC-DNA ELISA, mutation analysis of additional CRISPR/Cas-derived *Drosha*-deficient ES cell clones and DROSHA expression in *Drosha*-deficient ES cells transduced with *Drosha* cDNA. (**A**) Bar graphs indicate mean +/- standard deviation of replicate readings of DNA from wild type and *Drosha*$^{-/-}$ ES cells. (**B**) Sequence analysis of *Drosha* locus in the clones indicated. PAM is highlighted in red, modifications in each clone are shown. Data generated by MiSeq sequencing. (**C**) Western blot data using anti-Drosha antibody on RIPA cell lysates from cells indicated. Note absence of detectable Drosha protein in material from the various CRISPR/Cas-targeted clones. TKO indicates lysate from *Dnmt1*$^{-/-}$*Dnmt3A*$^{-/-}$*Dnmt3B*$^{-/-}$ ES cells. (**D**) Western blot using anti-Drosha antibody on RIPA lysates extracted from the ES cell lines indicated. Two independently transduced ES cell clones were analysed. Tubulin shown as a loading control.

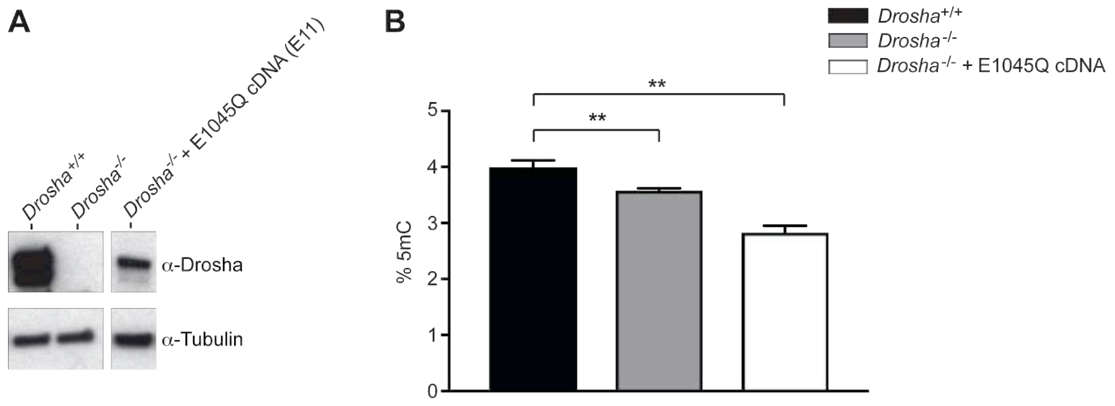

Supplementary Figure S9. Rescue of genomic hypomethylation requires Drosha catalytic activity. (**A**) Western blot using anti-Drosha antibody on RIPA lysates extracted from the ES cell lines indicated. Tubulin shown as a loading control. (**B**) Bar graphs showing LC-MS/MS results measuring percentage of 5-methyl-cytosine (5mC) in genomic DNA extracted from the cell lines indicated. Exact p-value = 0.0022 (Mann-Whitney test).

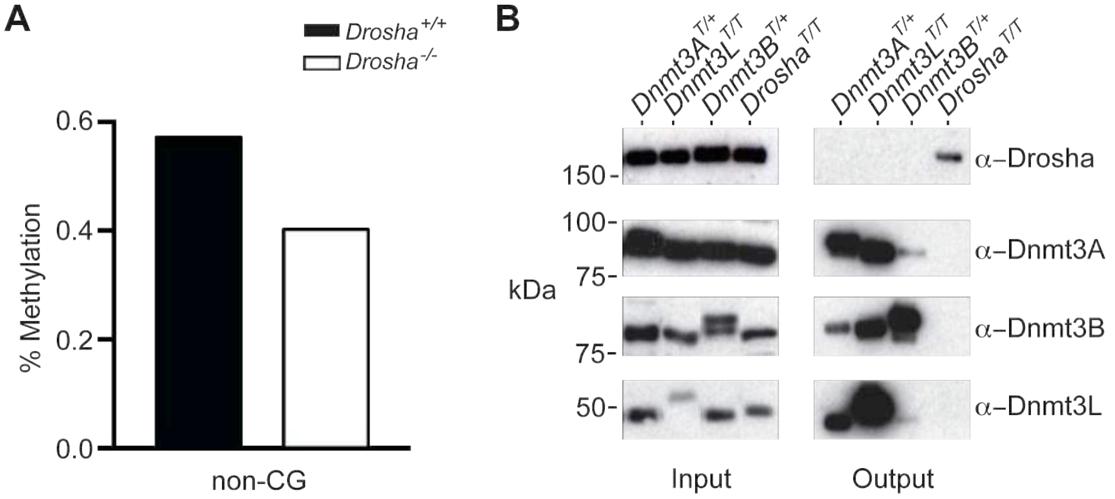

Supplementary Figure S10. Non-CG methylation is reduced in Drosha-deficient ES cells. (**A**) Bar graphs showing percentage cytosine methylation is either CG or non-CG contexts in wild-type and *Drosha*$^{-/-}$ ES cells. (**B**) Western blot using the antibodies indicated following immunoprecipitation with anti-FLAG M2 magnetic beads on material from tagged ES cell lines indicated. *T/+* and *T/T* indicates cells heterozygous or homozygous for c-myc-FLAG epitope tags, respectively.

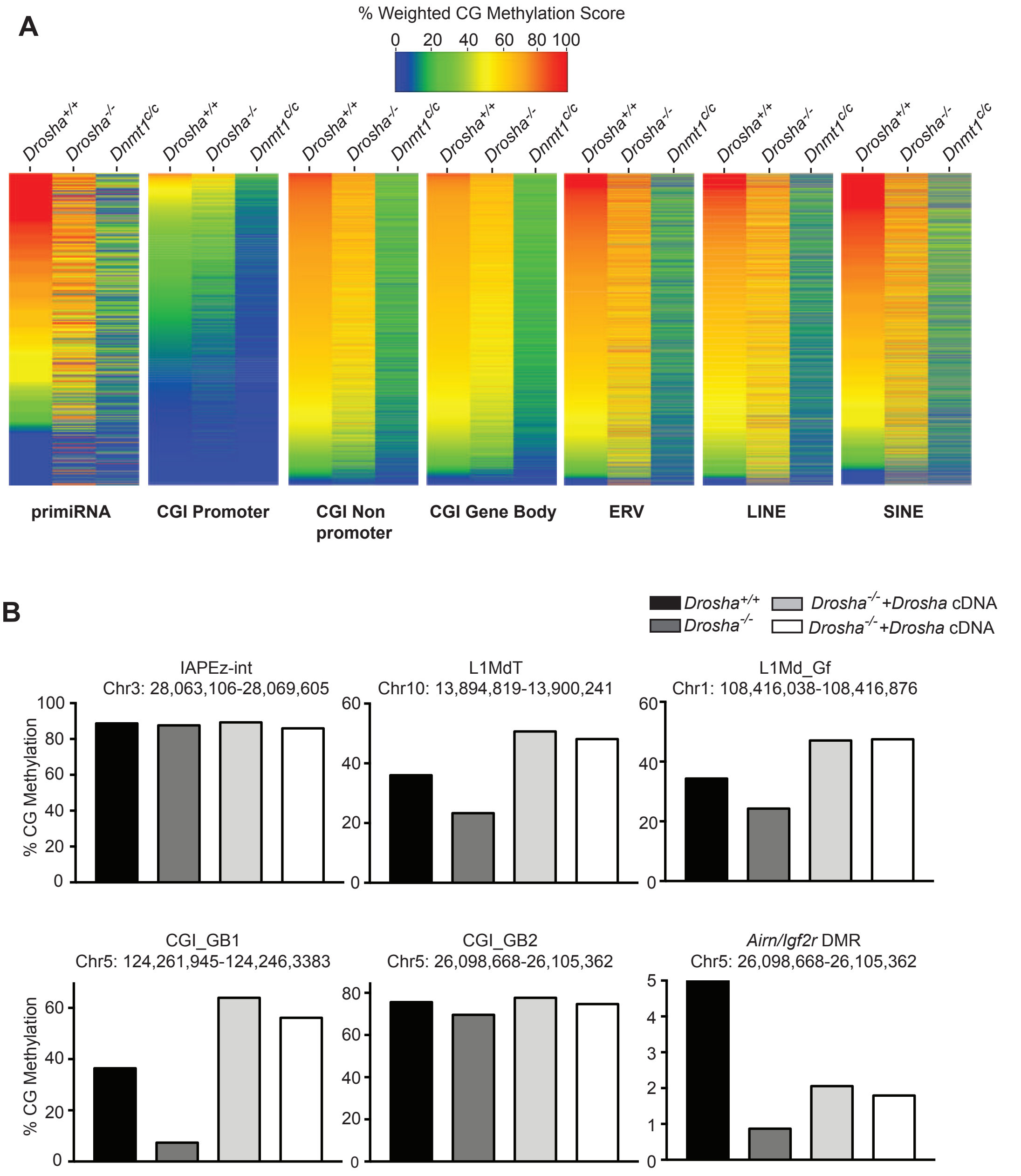

Supplementary Figure S11. Methylation analysis of different sequences in *Drosha*-deficient ES cells. (**A**) CG methylation heat maps generated from whole genome bisulphite sequencing (WGBS) data from DNA extracted from wild type, *Drosha*$^{-/-}$ and *Dnmt1*$^{c/c}$ ES cells. (**B**) Bar graphs showing data from Bisulphite Amplicon Sequencing (BSAS) of various target elements in the various cell lines indicated. Numbers of methylated and unmethylated reads at each CG within different elements are shown in Supplementary Table S2.

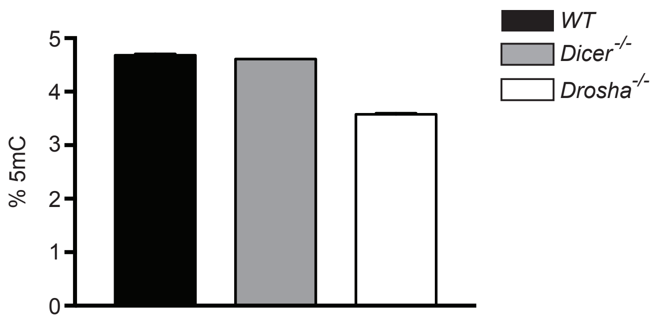

Supplementary Figure S12. Cytosine methylation analysis by LC-MS/MS in *Dicer*-deficient ES cells.

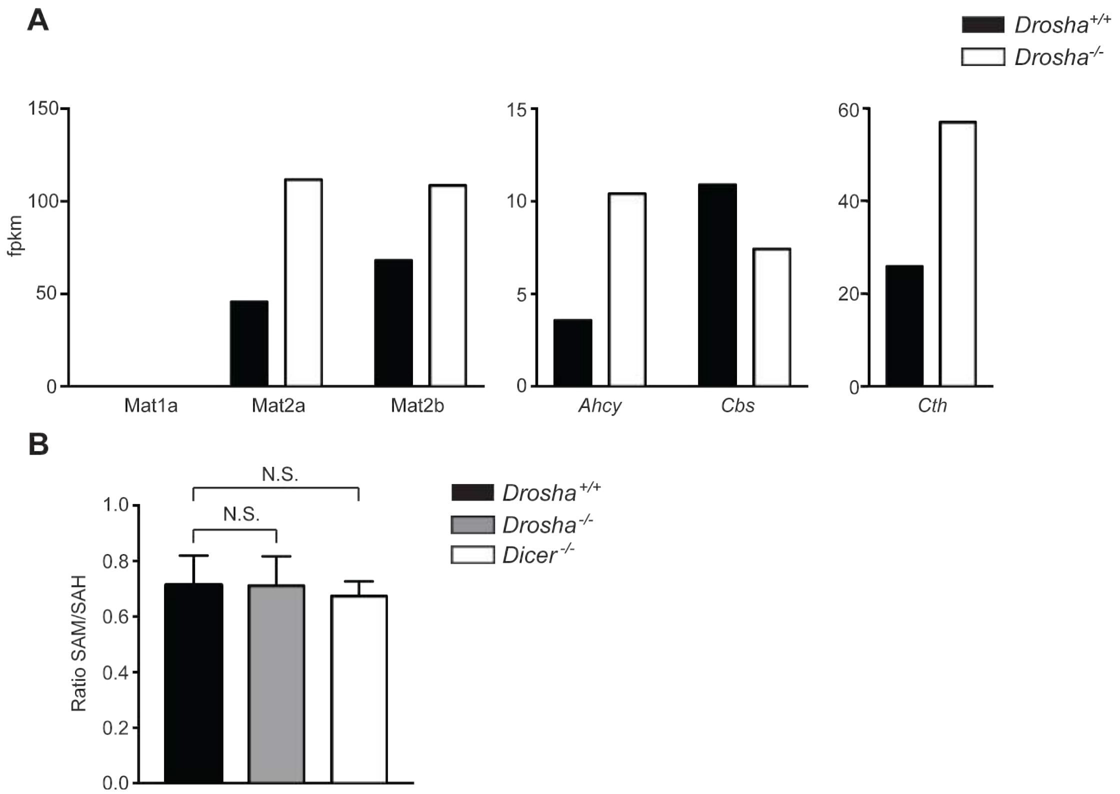

Supplementary Figure S13. Analysis of SAM-SAH metabolism. (**A**) Bar graphs showing fpkm from RNA-seq data of genes encoding enzymes involved in SAM metabolism. (**B**) Bar graphs showing ratio of SAM and SAH in the cell lines indicated. Exact Mann-Whitney P values are >0.9999 (wildtype vs. *Drosha*$^{-/-}$) and 0.6857 (wildtype vs. *Dicer*$^{-/-)}$.

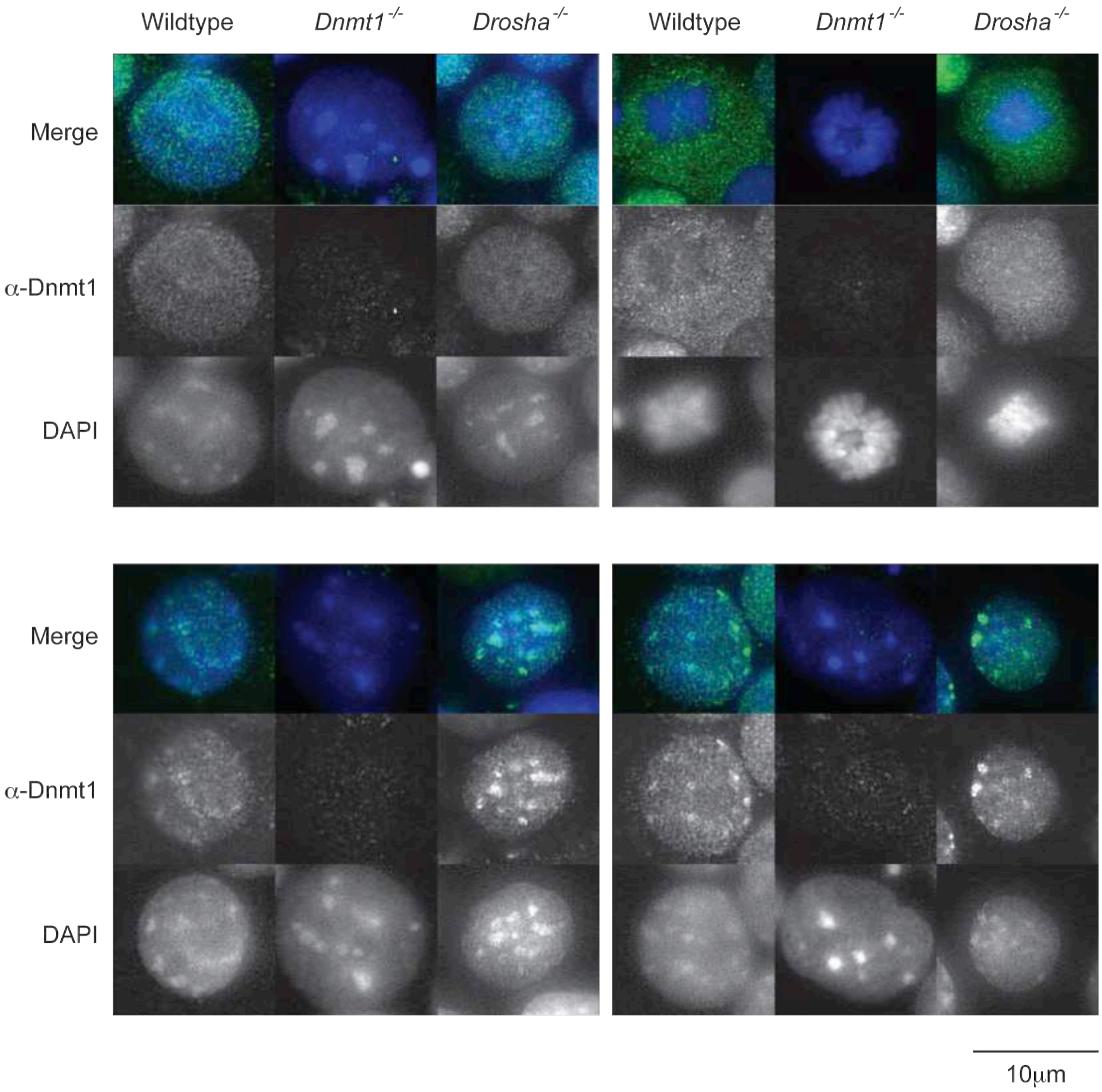

Supplementary Figure S14. Dnmt1 localisation is unaffected in Drosha-deficient ES cells. Immunofluorescence micrographs of ES cells with genotypes indicated stained with anti-Dnmt1 antibody. Nuclei are stained with DAPI.

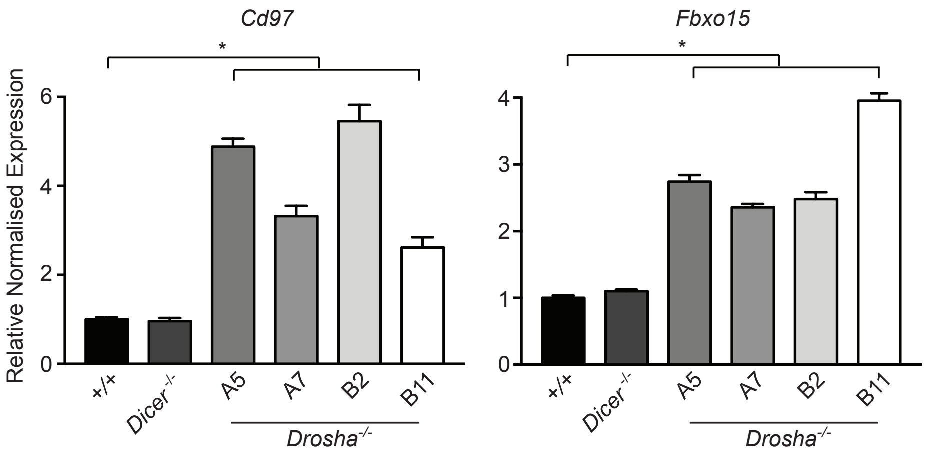

Supplementary Figure S15. *Cd97* and *Fbxo15* are upregulated in multiple *Drosha*-deficient ES cell lines. Exact p-value = 0.0286 (Mann-Whitney test). *Gapdh* used for normalisation.

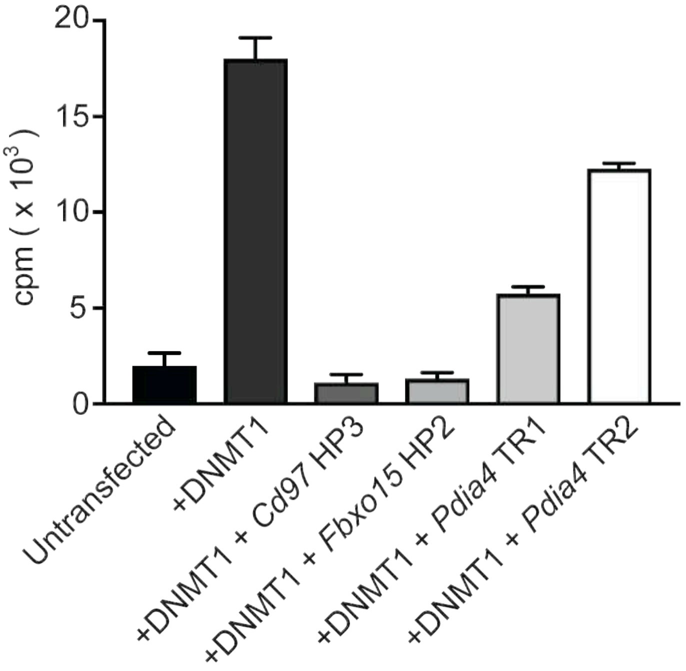

Supplementary Figure S16. *In vitro* maintenance methyltransferase activity is inhibited by RNA. Bar graphs indicate means +/- standard deviation of replicate readings. HP and TR indicate hairpin and target region, respectively.

Supplementary Table Figure Legends

Supplementary Table S1. small RNA-seq data showing miRNA expression in wildtype and *Drosha*-deficient ES cells.

Supplementary Table S2. Targeted methylation data from Bisulphite Amplicon Sequencing (BSAS).

Supplementary Table S3. List of primers used throughout this study.

Supplementary Methods

Generation of *Dnmt1* targeted allele

A synthetic oligonucleotide encoding the FLAG-c-myc epitope was introduced into the endogenous *Dnmt1* locus by homologous recombination in 129Sv/Ev ES cells (Millipore). Correct targeting was confirmed by Southern blot before removal of the *neo* cassette by transient expression of Cre recombinase. Southern blot, PCR and Sanger sequencing all confirmed correct excision of the *neo* cassette and presence of the FLAG-c-myc tag.

Immunofluorescence

ES cells were harvested and counted before cross-linking 2×10^6 in 1ml freshly prepared paraformaldehyde (2%, dissolved in PBS supplemented with calcium and magnesium) for 30mins on a Nutator at 4°C. Cells were then pelleted and washed in 1ml of PBS before re-suspension in 1ml 1x PBS, 1% goat serum, followed by further dilution (1:3) in 1x PBS+ 1% goat serum. 300 l aliquots were transferred onto glass slides using a Cytospin Cytocentrifuge (ThermoFisher Scientific) set at 800rpm for 4 minutes. Slides were then briefly air-dried for 5 seconds and transferred into Coplin jars containing 1 x PBS for 2-3 minutes. Slides were then transferred into Permeabilisation Solution (1 x PBS, 0.2% Triton X-100) for 12 minutes, before washing in 1 x PBS for 2-3 minutes followed by transfer into Block Solution (1 x PBS, 5% goat serum, 0.2% Tween-20) for 10 minutes. Anti-Dnmt1 antibody (PATH52) was prepared by dilution in Block Solution (1:500) and applied to cells on slides overnight at 4°C in a humidifying chamber. The next day, slides were rinsed in distilled water before incubation in 1 x PBS, 0.2% Tween-20 for 5 minutes. Slides were transferred to Block Solution for 10 minutes before applying anti-Rabbit- Alexa Fluor® 488-conjugated antibody diluted in Block Solution (1:1000) and incubated at room temperature in a darkened humidifying chamber. Slides were then rinsed in distilled water, incubated in 1 x PBS for 5 minutes before mounting in VECTORSHIELD Antifade Mounting Medium with DAPI (Vector Laboratories). Slides were analysed using a Zeiss Axio Observer Z1 (Zeiss).

Bisulphite Amplification Sequencing (BSAS)

Target methylation sites representing different regions were randomly selected and bisulphite PCR primers designed using MethPrime (1). Primers were designed to generate amplicons between 250 to 300 bp in size and included overhangs recognized by Illumina NeXTera transposons (Illumina, CA). DNA from various cell lines indicated was bisulphite converted using EZ DNA Methylation-Lightning Kit (Zymo Research, CA) according to the manufacturer's protocol. PCRs were performed using ZymoTaq™ Polymerase (Zymo Research, CA) using primers indicated in Appendix Table S3. Note, for the purpose of library construction, all primer sequences included a universal tag (5'-TCGTCGGCAGCGTCAGATGTGTATAAGAGACAG-3 for forward primers and 5'-GTCTCGTGGGCTCGGAGATGTGTATAAGAGACAG-3' for reverse primers). Following PCR, amplicons were resolved by agarose gel-electrophoresis, excised and DNA was purified using QIAquick Gel Extraction Kit (Qiagen). Following quantification by Nanodrop (Thermo Fisher Scientific), 100 to 125ng of each amplicon was subjected to limited cycle number PCR using Kapa HiFi Hot Start Polymerase (Kapa Biosystems). PCR amplicons were resolved by agarose-gel electrophoresis before excision and purification using Mini-Elute Gel Extraction kit (Qiagen). Samples were quantified before sequencing on a MiSeq Sequencer (Illumina, CA). Methylation analysis was performed using Bismark (2).

Identification of putative off-target sites

Putative off target sites were identified using WTSI Genome Editing website (http://www.sanger.ac.uk/htgt/wge/). PCR primers flanking potential Cas9 cut sites were used to amplify each locus and analyzed by either Sanger or MiSeq sequencing.

SAM/SAH ratiometric analysis

Cell lysates were prepared from freshly harvested ES cells. Aliquots of 10 million cells were re-suspended in 1ml of 1 x PBS and sonicated using a Sonicator® 3000 Ultrasonic Liquid Processor (QSonica) fitted with a microtip horn (power setting 1.0, 12 pulses of 15 seconds with 10 second recovery periods between each pulse and mixing after every third pulse). Lysis was confirmed by visual inspection under a light microscope. Lysates were clarified by centrifugation at 10,000 rcf for 10 minutes. Supernatant was transferred into fresh tubes and the samples flash-frozen and stored at -80°C. Samples were quantified using DC Protein Assay (Biorad) and equal amounts (18μg) of lysates was used for analysis. SAM/SAH levels were determined using S-Adenosylmethionine (SAM) and S-Adenosylhomocystenine (SAH) ELISA Combo Kit (Cell Biolabs) according to the manufacturer's protocol. Absorbance at 450nm was measured using a Thermo Scientific Multiskan FC and Skanit™ software (Thermo Scientific).

References

1. Li, L.C. and Dahiya, R. (2002) MethPrimer: designing primers for methylation PCRs. *Bioinformatics*, **18**, 1427-1431.
2. Krueger, F. and Andrews, S.R. (2011) Bismark: a flexible aligner and methylation caller for Bisulfite-Seq applications. *Bioinformatics*, **27**, 1571-1572.
